# Revolutionizing bio-hydrogen production: smart integration of nanotechnology, microbial engineering, and circular waste valorisation

**DOI:** 10.1186/s40643-025-01001-4

**Published:** 2026-01-28

**Authors:** Krishna Chaitanya Maturi, Siva Rama Krishna Madeti, Sanjana Sinha, Silvia Saikia, Abhishek Srivastava, Izharul Haq

**Affiliations:** 1 Civil Engineering, School of Technology, Gati Shakti Vishwavidyalaya (A Central University Under the Ministry of Railways, Government of India), Vadodara, Gujarat 390004 India; 2 Electrical Engineering, School of Technology, Gati Shakti Vishwavidyalaya (A Central University Under the Ministry of Railways, Government of India), Vadodara, Gujarat 390004 India; 3 School of Agro and Rural Technology, Indian Institute of Technology Guwahati, Kamrup, Assam, 781039 India; 4 Department of Civil Engineering, National Institute of Technology Calicut, Kozhikode, Kerala 673601 India; 5 Department of Biosciences, Manipal University Jaipur, Jaipur, Rajasthan 303007 India

**Keywords:** Bio-hydrogen revolution, Nano-bioengineering, Circular bio-economy, Waste-to-energy valorization, Smart microbial systems, Sustainable energy transformation

## Abstract

The escalating depletion of fossil fuel reserves and mounting environmental concerns from greenhouse gas emissions have intensified the global pursuit for sustainable energy alternatives. Bio-hydrogen production emerges as a transformative solution, offering carbon–neutral energy generation while simultaneously addressing organic waste management challenges. This comprehensive review examines the revolutionary integration of nanotechnology, advanced microbial engineering, and circular economy principles in bio-hydrogen production systems. A systematic analysis of diverse renewable feedstocks, including agricultural residues, municipal solid waste, microalgae, and industrial biomass, highlighting their potential for decentralized bio-hydrogen production. The review critically evaluates cutting-edge microbial innovations encompassing hybrid fermentation systems, extremophile consortia, and synthetic biology approaches utilizing CRISPR-Cas9 technology for enhanced hydrogen yields. Nanotechnology applications are extensively discussed, focusing on nano-metal catalysts, enzyme immobilization techniques, and plasmonic nanoparticles that significantly improve bioconversion efficiency and system stability. Advanced purification technologies, including mixed-matrix membranes and graphene-based systems, alongside innovative storage solutions using metal hydrides, are comprehensively assessed. The integration of bio-hydrogen into fuel cells and industrial applications demonstrates substantial potential for replacing fossil-based hydrogen. This review establishes bio-hydrogen as a cornerstone technology for achieving sustainable energy transitions while fostering circular bio-economy development.

## Introduction

The rapid depletion of fossil fuel reserves, combined with the increasing level of deleterious greenhouse gas emissions, has driven the global search for alternative, renewable energy sources. Fossil fuels such as oil and coal, accounting for an estimated 80% of the world’s energy supply, have not only led to a depletion of natural resources but have also precipitated a continual rise in atmospheric carbon dioxide levels, with figures recently noted at roughly 407.8 ppm (Welsby et al. 2021). In this context, bio-hydrogen production has emerged as a promising green technology, particularly due to its potential role in both reducing greenhouse gas emissions and mitigating the adverse impacts of climate change (Friedlingstein et al. 2025).

A growing body of research has turned its attention to microalgal biomass as a renewable feedstock that overcomes many of the limitations inherent in using terrestrial crops. Microalgae, due to their high growth rates, efficient photosynthesis, and the ability to thrive in diverse and even extreme habitats (including fresh, marine, and marginal lands), are positioned as an ideal candidate for biofuel production (Khan et al. 2018; Wang et al. 2024). Unlike terrestrial crops that compete with food production, microalgae can be cultivated on non-arable lands and require only minimal nutrient inputs while also sequestering atmospheric carbon dioxide during growth, a dual benefit that ties energy production directly to environmental remediation (Hosny et al. 2025).

In the realm of bio-hydrogen generation, the microalgal route is particularly attractive because various physiological conditions, such as oxygen deprivation combined with high light exposure, can spur cells to produce hydrogen. Despite these promising attributes, the underlying mechanisms by which microalgae generate hydrogen are diverse and at times sporadic. Established processes include biophotochemical pathways, fermentation, bioelectrochemical methods, and the more recent approaches based on biotechnology and genetic engineering (Jiao et al. 2024). Nonetheless, these mechanisms have not been exhaustively documented, and the factors affecting the actual yield of hydrogen remain poorly understood. This knowledge gap has so far limited the scale and efficiency of bio-hydrogen production, necessitating further research into both the biology and the engineering of these microalgal systems.

Technologically, the unique attributes of microalgae provide several significant advantages over other biomass sources. Their ability to grow on non-arable land and in diverse habitats means that they do not exacerbate the common “food versus fuel” dilemma associated with many crop-based biofuels. Moreover, the implementation of state-of-the-art techniques, such as advanced genetic engineering to optimize microalgal strains, promises to enhance hydrogen yields, thereby making the process more viable at an industrial scale (Zainal et al. 2024). This opens up the potential for integrating bio-hydrogen production with a broader bio-refinery concept, wherein the residual biomass can be utilized to produce a suite of co-products (e.g., bioethanol, biodiesel, and biogas). Such integration not only improves the economic feasibility of these processes but also fosters a circular economy approach in renewable energy sectors.

Another area of burgeoning significance is the application of hydrogen in the transportation sector. Hydrogen-powered transportation, including its prospective use in aviation, could herald a paradigm shift away from traditional hydrocarbon fuels. The use of hydrogen in jet engines, for example, would result in the emission of pure water vapour rather than carbon-based emissions, representing a major leap forward in sustainable air travel (Hosny et al. 2025; Welsby et al. 2021). However, this shift demands overcoming practical hurdles such as redesigning aircraft to accommodate larger storage tanks and developing robust leak detection systems. Therefore, research in microalgal bio-hydrogen not only directly addresses environmental and energy concerns but also pushes the boundaries of existing engineering practices to adapt conventional transportation systems to new renewable energy models.

Building upon these technological foundations, the integration of nanotechnology with microbial systems represents a revolutionary advancement in bio-hydrogen production, fundamentally transforming the efficiency and viability of biological hydrogen generation processes. Recent research has shown evidence of the promising capabilities of nano-catalysts and nanomaterials in enhancing the hydrolysis of lignocellulosic biomass, facilitating sugar synthesis, and improving the conversion of biomass into bioenergy (He et al. 2022). In microbial electrolysis cells (MECs), nanomaterial integration has demonstrated remarkable improvements in bio-hydrogen production rates. Graphite electrodes decorated with Au nanoparticles gave up to 20 times more hydrogen production compared to normal graphite electrodes, while TiO_2_ nanotubes used as catalysts for photo-anodes improved MECs efficiency to 1434 mmol/m^3^/h compared to dark conditions (Abd-Elrahman et al. 2022). The unique properties of nanomaterials, including their crystalline nature, stability, adsorption ability, catalytic properties, increased electroconductivity, and high surface-to-volume ratio, significantly enhance hydrogen generation by overcoming metabolic pathway limitations that typically shift toward other products (El-Sheekh et al. 2025). Furthermore, carbon nanotubes have emerged as particularly effective anode materials due to their excellent electrical, mechanical, biological, and thermal properties (Kumar et al. 2025).

Advanced nanocomposites, such as reduced graphene oxide (rGO) nanosheets combined with CeO_2_ nanoparticles, have achieved cathode bio-hydrogen recovery rates of 98% by facilitating efficient electron transfer processes (Liu et al. 2020). The electro-deposition of nickel nanoparticles (30–50 nm) on carbon paper cathode electrodes has shown superior catalytic performance compared to platinum while significantly reducing operational costs (Figueroa-Torres et al. 2012). Photo Nano catalysts feature large band gaps and high defect concentrations, thus having the ability to be tuned for their characteristics, enabling customization for enhanced photo-fermentative bio-hydrogen production from biomass wastes (He et al. 2022). These technological advances demonstrate that nanotechnology integration addresses fundamental bottlenecks in bio-hydrogen production by enhancing enzyme stability, improving electron transfer efficiency, and optimizing the overall bio-electrochemical performance of microbial systems.

The synergistic combination of nanotechnology with advanced microbial engineering techniques has opened unprecedented pathways for optimizing bio-hydrogen production through multi-faceted enhancement strategies. The integration of nanoparticles into substrates to enhance bio-hydrogen production is considered a robust approach, with magnesium identified as one of the essential cofactors that activate more than ten enzymes involved in hydrogen fermentation (Cheng et al. 2022). Recent developments in nano biotechnology have focused on enzyme immobilization techniques, where nanoparticles serve as carriers for hydrogenase and nitrogenase enzymes, protecting them from oxygen sensitivity while maintaining their catalytic activity (Sekoai et al. 2019).

The application of microalgae-based nanotechnology for efficient and sustainable bioremediation processes has shown desirable properties, including tunable surface functions, structural stability, high adsorption capabilities, improved selectivity and specificity, enhanced biodegradability, and increased reusability (Cheng et al. 2022). Magnetic nanoparticles have been particularly effective in creating recoverable enzyme systems, allowing for continuous bio-hydrogen production while maintaining enzyme integrity over extended operational periods (Ladole et al. 2017). The development of hybrid nano-bio systems has also addressed the challenge of metabolic pathway optimization, where engineered nanocarriers deliver genetic modification tools directly to target microorganisms, enhancing their hydrogen-producing capabilities (Srivastava et al. 2020). Advanced MECs with the presence of nanoparticles have demonstrated enhanced biotransformation properties, with interactions between nanoparticles, bacteria, substrates, and enzymes significantly improving bio-hydrogen production rates (Arun et al. 2024).

Furthermore, the application of nano-biocatalysts in photo-fermentation systems has shown promising results in overcoming light penetration limitations and photo-inhibition effects that typically reduce hydrogen yields (Giannelli and Torzillo 2012). Recent studies have demonstrated that silver and gold nanoparticles can serve as electron mediators, facilitating more efficient electron transfer from photosynthetic reactions to hydrogen-producing enzymes (Mousavi et al. 2019). The future of nanobiotechnology in bio-hydrogen production lies in developing smart, responsive nano systems that can adapt to changing environmental conditions, self-regulate enzymatic activities, and provide real-time monitoring capabilities for optimal hydrogen production efficiency. These integrated nano-bio systems represent the next generation of sustainable energy technologies, bridging the gap between laboratory-scale research and industrial-scale implementation of clean hydrogen production from renewable microalgal feedstocks.

### Motivation and novelty of the review

Despite the rapid growth of literature on hydrogen production, most existing reviews remain fragmented, focusing either on conventional biological processes, individual feedstocks, or isolated nanotechnology applications. The contribution of this review lies in its integrated framework that unites microalgae-based renewable feedstocks, microbial innovations, and nanotechnology-driven enhancements into a single roadmap for sustainable bio-hydrogen production. It addresses critical gaps such as limited documentation on the synergistic role of nanoparticles in overcoming metabolic and electron transfer bottlenecks, inadequate emphasis on hybrid feedstocks that resolve the food–fuel conflict, and a lack of clear strategies for bridging laboratory-scale breakthroughs with industrial deployment. By systematically linking biological, technological, and circular economy dimensions, this review contributes a forward-looking perspective that not only consolidates current knowledge but also identifies unexplored directions and interdisciplinary solutions essential for scaling bio-hydrogen production into real-world applications.

### Role of renewable feedstock in energy sustainability

The transition toward a carbon–neutral future is increasingly dependent on renewable resources capable of meeting global energy demands without exacerbating environmental degradation (Maturi et al. 2022a). Among these, bio-hydrogen emerges as a key sustainable energy vector, with its production intricately linked to the utilization of renewable and waste-derived feedstocks. Agricultural residues, microalgae, municipal solid waste, and industrial biomass offer unique advantages: high biodegradability, rich carbohydrate content, low-cost availability, and minimal land-use conflicts (Maturi et al. 2022b).

These feedstocks not only eliminate the “food vs. fuel” challenge but also enable waste valorization by integrating energy recovery with environmental management. Microalgae, for instance, not only serve as an excellent biomass source for hydrogen production but also act as natural carbon sinks, further enhancing their ecological relevance. Similarly, organic waste streams such as food waste and sewage sludge, when converted into hydrogen through biological or thermochemical pathways, present a decentralized, resource-efficient route to green bio-hydrogen production.

**Fig. 1:**
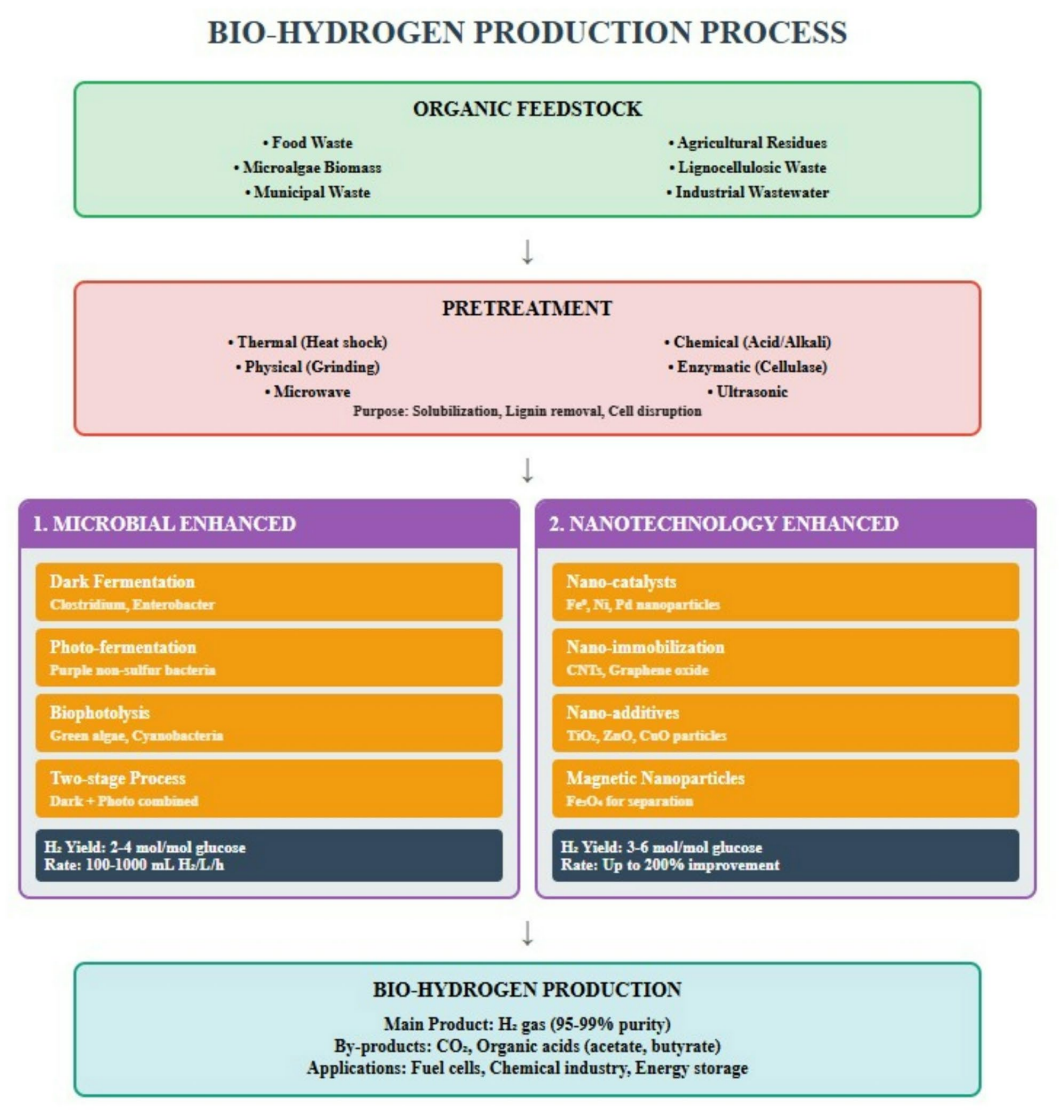
A flowchart showing the whole process of bio-hydrogen production with microbial and nanotechnology enhancement

The sustainability of these systems is further amplified through circular economy practices, where residual biomass and process by-products are recovered as value-added materials, fertilizers, or co-substrates for other bio-processes. Therefore, renewable feedstocks are not just the input materials for hydrogen production but are enablers of a broader ecosystem of environmental remediation, energy decentralization, and circular resource utilization. Table 1 depicts a brief review of feedstock utilization, scale, and technology of bio-hydrogen production. Figure 1 depicts the flowchart showing the whole process of bio-hydrogen production with Microbial and nanotechnology enhancement.

**Table 1:** Brief literature on feedstock utilization, scale and technology of bio-hydrogen production

S. no	Feedstock used	Composition (Cellulose/Hemicellulose/Lignin; COD)	Pretreatment Used	Scale	Bio-hydrogen production technology (DF/PF/Gasification/MEC)	H_2_ Yield	Optimal conditions	Inhibitors/limitations	References
1	Food waste	High carbohydrates, proteins, lipids; COD 80,000–120,000 mg/L	Heat-shock + homogenization	Laboratory	Dark fermentation	2.1–2.8 mol H_2_/mol substrate	pH 5.5–6.0; 37 °C	VFA accumulation, ammonia inhibition	Okoro-Shekwaga and Wilmshurst (2024)
2	Corn stover	Cellulose 35–45%, hemicellulose 25–30%, lignin 15–20%	Acid/alkali + enzymatic hydrolysis	Pilot	Integrated DF + PF	3.0–3.4 mol H_2_/mol glucose equivalent	30–35 °C; pH 6.0	Recalcitrant lignin; expensive pretreatment	Zhang et al. (2024)
3	Agro-industrial waste	Variable composition: sugars 20–40%, proteins 15–25%	Acid + thermal pretreatment	Laboratory	Dark fermentation	1.5–2.2 mol H_2_/mol substrate	Mesophilic range	High salinity, presence of phenolics	Usman et al. (2019)
4	Agricultural residues	Cellulose 35–50%, hemicellulose 20–30%, lignin 10–20%	Milling + dilute acid	Laboratory	Two-stage dark fermentation	2.5–3.1 mol H_2_/mol glucose	pH 5.5; 55 °C (thermophilic)	Solid–liquid mass transfer limitations	Massuque et al. (2023)
5	Microalgae–bacterial consortium	High protein 30–55%; carbohydrates 20–40%	Mild thermal + enzymatic	Laboratory	Photo-fermentation	1.3–1.8 L H_2_/L	Light intensity 4–6 klux	Light sensitivity; oxygen inhibition	Iqbal et al. (2022)
6	Microalgae	Carbohydrates 20–50%; protein 30–60%	Cell-wall disruption	Laboratory	Multiple pathways (biophotolysis, PF, DF)	1.0–2.5 mol H_2_/mol	Light-dependent; pH 7–8	O_2_ sensitivity of hydrogenase	Li et al. (2022a, b)
7	Organic feedstocks	COD 50,000–150,000 mg/L	Various (reviewed)	Review study	Dark fermentation	—	—	—	Dahiya et al. (2021)
8	Barley straw	Cellulose 38–42%, hemicellulose 22–28%, lignin 15–18%	Alkali pretreatment	Laboratory	*Saccharomyces cerevisiae* fermentation	0.8–1.3 mol H_2_/mol hexose	30 °C; pH 6–7	Low H_2_ yield due to yeast metabolism	Malik et al. (2020)
9	Vegetable by-products	Sugars 10–25%, cellulose 15–20%, proteins 8–12%	Mechanical + enzymatic	Laboratory	Lactic acid bacteria fermentation	0.4–0.8 mol H_2_/mol	35 °C; pH 6.5	LAB favor lactate—not H_2_ production	Sabater et al. (2020)
10	Green organic waste	Cellulose ~ 35%, hemicellulose 25%, lignin 15%	Shredding + heat treatment	Laboratory	Microbial fermentation	1.5–2.0 mol H_2_/mol	37 °C; pH ~ 6	Low solubilization; heterogeneity	Yang et al. (2019)
11	Pretreated microalgal biomass	High protein and carbohydrate	Thermal–acid pretreatment	Laboratory	Dark fermentation	1.6–2.1 mol H_2_/mol	35–40 °C; pH 6	Ammonia inhibition	Wang and Yin (2018)
12	Wheat straw	Cellulose 38–45%, hemicellulose 28–32%, lignin 15–18%	Dilute acid pretreatment	Laboratory	*E. coli* fermentation	0.9–1.5 mol H_2_/mol	30–37 °C	Low H_2_ yield due to metabolic constraints	Lopez-Hidalgo et al. (2017)

### Objectives of the review

This review aims to examine the current advancements in bio-hydrogen production processes, encompassing biological, thermochemical, and electrochemical methods. It highlights the role of renewable biomass and organic waste as sustainable feedstocks for bio-hydrogen generation, emphasizing their potential to support a circular and low-carbon energy framework. The study further explores how nanotechnology can enhance hydrogen yield, catalyst performance, and overall system efficiency across both production and storage stages. In addition, it analyses microbial innovations such as hybrid fermentation and genetic engineering approaches that improve bio-hydrogen productivity and operational reliability. Finally, the review assesses the integration of bio-hydrogen into real-world applications, particularly focusing on its deployment in decentralized energy systems and industrial sectors.

### Scope of the review

The review spans the entire bio-hydrogen value chain, encompassing feedstock selection, microbial processing, purification, storage, and end-use applications. It includes an evaluation of nanomaterial-based enhancements in enzymatic, catalytic, and membrane processes that contribute to improved system performance and efficiency. Furthermore, the review covers both laboratory-scale breakthroughs and scalable industrial models, emphasizing the principles of the circular economy and waste-to-energy concepts. It also investigates policy-relevant challenges such as regulatory requirements, safety standards, and cost barriers that hinder large-scale deployment. Finally, the paper outlines future research directions, proposing technological and interdisciplinary solutions to facilitate the widespread adoption of bio-hydrogen.

## Bridging energy and waste management

### Global perspectives on biohydrogen: potential, limitations, and challenges

The hydrogen is becoming a potent component in the worldwide clean energy transition due to its high heating value and zero emissions during its application. The need for hydrogen is predicted to increase in the sectors of industrial, power production, and transportation, according to the International Energy Agency (IEA) (Hassan et al. 2024). However, the fossil fuel-based hydrogen (grey and blue hydrogen) leads the existing hydrogen economy, denying the environmental benefits of hydrogen (Vezzoni 2024). An initiative towards green hydrogen derived from renewable resources is highlighted by current international programs.

An ecologically valuable alternative is bio-hydrogen, which is manufactured by biological processes such as microbial electrolysis, dark fermentation, and photo-fermentation (Senthil Rathi et al. 2024). It offers the benefit of integrating the treatment of organic waste with bio-hydrogen production, supporting sustainable development objectives. The potential for accessible renewable bio-hydrogen production was underlined in literature, which demonstrated that bio-hydrogen synthesis utilizing municipal solid waste may attain yields of over 3.2 mol H_2_/mol glucose equivalent (Obiora et al. 2024).

The application of bio-hydrogen has been included in national hydrogen initiatives in several nations. South Korea and Japan, for example, have made investments in bio-hydrogen pilot projects that employ food waste and agricultural waste (Cappai et al. 2018). Under the Clean Hydrogen Partnership, the European Union’s Horizon Europe program also sponsors bio-hydrogen research through programs like BioH_2_Cluster, which examines decentralized bio-hydrogen production plants employing bio-waste (European Commission, 2023). To upsurge sustainability, hybrid systems that combine the generation of bio-hydrogen with carbon capture and storage (CCS) are also being studied (Kazmi et al. 2024). When compared to typical hydrogen generation, such systems may save lifecycle greenhouse gas emissions by up to 85% (J. Li et al. 2022a, b). Furthermore, bio-hydrogen is becoming more and more viewed as important for businesses where electrification is less practicable, such as heavy transportation and industrial heating. Overall, as regulatory frameworks and technical improvements converge to promote low-carbon hydrogen sources, bio-hydrogen’s position in the global hydrogen economy is expanding in importance. Bio-hydrogen possibly will play an important role in enabling the change to a circular and decarbonized energy future with greater investigation and financing.

The universal shift to sustainable energy is triggering more research on the methods of generating hydrogen. The production of hydrogen using fossil fuels leads to the emission of greenhouse gases (GHG) (Pivac et al. 2024). Life cycle assessment (LCA) studies show that grey hydrogen generated without carbon capture releases between 12.3 and 13.9 kg CO_2_-equivalent per kg of hydrogen. Including CCS, even the emissions from the generation of blue hydrogen are about 7.6 to 9.3 kg CO_2_-equivalent per kg of hydrogen (Patel et al. 2024). On the other hand, the production of bio-hydrogen by fermentation and biomass gasification exhibits lower greenhouse gas emissions. Using advanced gasification technologies, such as bio-hydrogen generated from municipal solid waste (MSW), has shown net-negative carbon emissions, thereby essentially sequestering more CO_2_ than it releases (Lefranc et al. 2024). This ability makes bio-hydrogen a potent means for reducing the consequences of global warming. Water utilization is yet another crucial factor influencing the viability of hydrogen generation. The cultivation of biomass and processing for the production of hydrogen demands a lot of water, resulting in higher water footprints. However, the water footprint can be reduced by feeding organic leftovers or waste biomass, enhancing the overall sustainability of bio-hydrogen (Olaitan et al. 2024). The implementation of bio-hydrogen production requires overcoming challenges such as infrastructure development, technological scalability, and process efficiency (Meena and Patane 2025). Recent studies show the application of nanotechnology to boost the efficiency of bio-hydrogen generation. It has been established that utilizing photoactive nanoparticles and catalysts can increase process stability and yields of hydrogen (Li et al. 2024). Thus, the reduction of carbon footprint makes bio-hydrogen a more viable choice than hydrogen obtained from fossil fuels.

### Waste-to-hydrogen: transforming waste into green energy

The conversion of waste into hydrogen is a promising development in clean energy production and sustainable waste management as a “waste-to-hydrogen” technology. The high energy content of organic waste, such as food waste, sewage sludge, MSW, and agricultural residues, produces a high-energy hydrogen fuel that emits no carbon dioxide when burned (Tian et al. 2019).

#### Types of waste potential for biohydrogen production

The biohydrogen derived from organic waste directs towards waste minimization in the landfill, reduction of greenhouse gas emissions, and energy recovery. To develop a sustainable biohydrogen technology, a comprehensive understanding of the type of waste, its management, pre-treatment, and valorization techniques is crucial. Agricultural residues such as rice and wheat straw, corn cobs, maize stalk, sugarcane bagasse, and agro-industrial by-products such as molasses and oilseed cakes constitute a significant biomass resource (Mujtaba et al. 2023). These wastes are rich in lignocellulose, which often ends up in open burning or open dumping rather than its potential utilization. Forestry and wood processing waste, such as sawdust, wood chips, twigs, and tree barks, are potential lignocellulosic materials, high in cellulose and hemicellulose, thus providing fermentable sugars after pretreatment (Bueno Moron et al. 2025). Municipal food waste, leftover food from restaurants, and household kitchen waste are often mismanaged, leading to landfill accumulation, methane emissions, and leachate generation, even though they are rich in carbohydrates and proteins.

The organic fraction of municipal solid waste, which includes vegetable peels, fruits, garden sweepings, and waste paper, has major potential for hydrogen recovery. These wastes are mostly treated by composting or dumped in landfills, which can also be redirected towards biohydrogen production (Liu et al. 2024). Industrial waste and effluents from starch processing, dairy, distillery, brewery, and sugar industries produce high-strength organic effluents containing soluble sugars and proteins, which are highly suitable for dark fermentation (Ghimire et al. 2015b). Sewage sludge and manure from livestock are abundant in organic matter and nutrients. The anaerobic fermentation of these wastes offers a route to valorize these wastes while reducing environmental hazards (Parra et al. 2025).

At present, waste management generally relies on landfilling, composting, anaerobic digestion, or incineration. These methods are effective for volume reduction but not optimal for hydrogen recovery. The integration of waste-to-biohydrogen technologies into existing solid waste management systems provides dual benefits of waste treatment and clean bio-hydrogen production. An effective segregation of organic fractions, collection systems, and preprocessing steps is fundamental for efficient hydrogen production.

#### Pre-treatment and valorization of waste for biohydrogen production

The conversion of organic waste into hydrogen is generally done by thermochemical and biological processes. Gasification and pyrolysis are two of the most common thermochemical processes. The partial oxidation of biomass at high temperatures (usually 800–1000 °C) produces syngas, which is a mixture of hydrogen, carbon monoxide, and methane (Canabarro et al. 2013).

The biohydrogen production from various waste is generally inhibited by the recalcitrant characteristics of biomass and the heterogeneous nature of the waste. To convert biomass or waste and enhance the yield of hydrogen efficiently, it requires methodical pre-treatment, valorization, and careful selection of the production routes. Biomass and organic wastes generally have a structure of complex polymers, such as cellulose, hemicellulose, lignin, proteins, and lipids, that are not directly accessible for microbial hydrogen generation (Kamdem Tamo et al. 2025). Therefore, pretreatment methods are necessary for the breakdown of the structural barriers and improvement of biodegradability. Physical treatment, such as milling, grinding, ultrasonication, and hydrothermal treatment, enhances the surface area and solubilizes organic fractions (Sant’Ana Júnior et al. 2025).

The chemical treatment of the substrate through acid, alkali, ozonation, and ionic liquid breaks down the lignocellulosic structures, which releases fermentable sugar. However, if the chemical treatment process is not optimized, then it may produce inhibitory compounds (Behera et al. 2014). The biological pre-treatment includes microbial or enzymatic hydrolysis that degrades lignin and polysaccharides selectively. The energy requirement in this process is less but comparatively slower than the physicochemical processes. However, to maximize the efficiency, reduce inhibitor formation, and improvement of cost-effectiveness of biohydrogen production, a combined method of thermochemical and chemoenzymatic process is implemented (Chen et al. 2024).

The valorization of waste for biohydrogen production not only converts waste to biohydrogen but also generates value-added intermediates and byproducts. Organic waste such as food waste, agro-industrial waste, organic fraction of municipal solid waste, and wastewater sludge are feedstock without any cost. The valorization of these wastes reduces the environmental impact, simultaneously providing a renewable energy source. The co-product, such as organic acids, biochar, and biopolymer recovery during biohydrogen production, boosts economic feasibility. For example, the volatile fatty acids produced during fermentation can be upgraded to bioplastics or can be used in chain elongation to produce higher-value fuels (Bevilacqua et al. 2022; Jin et al. 2025). The integration with the circular economy confirms the maximum utilization of resources by connecting hydrogen production with nutrient recovery, such as nitrogen, phosphorus, and potassium, and carbon management (Ates et al. 2025). To purify the hydrogen and increase overall yields, sophisticated gas cleaning and reforming procedures are necessary. The high conversion efficiencies and scalability make thermochemical routes beneficial. However, the high energy consumption, sophisticated reactor, and gas-cleaning systems lead to a costlier technology (Rey et al. 2024).

##### Routes to biohydrogen production

There are many biological pathways available for the conversion of valorized as well as pretreated waste into biohydrogen. To adopt the process for production, it is required to determine the composition of feedstock, the yield quantity, and the integration of the process.

- Dark Fermentation (DF): In this route, anaerobic bacteria such as *Clostridium* and *Enterobacter* convert carbohydrates into hydrogen and organic acids (Sim et al. 2023). Even though yields of biohydrogen are typically limited by thermodynamic limitations, DF is attractive due to its simplicity, rapid conversion, and ability to process diverse substrates.
- Photofermentation (PF): In this method, photosynthetic bacteria such as *Rhodobacter* and *Rhodopseudomonas* utilize organic acids mostly from DF effluents under light to generate additional hydrogen, offering a two-stage synergistic route (Wu et al. 2010).
- Biophotolysis: In the process of biophotolysis, microalgae and cyanobacteria dissociate water directly into hydrogen and oxygen using light energy through [NiFe]-hydrogenases. In this route, the challenge is that the yield of biohydrogen is low and is oxygen sensitive. However, this method has the potential for direct solar-to-fuel conversion (Allakhverdiev et al. 2010).
- Hybrid systems: Integrating DF with PF, microbial electrolysis cells (MECs), or thermochemical processes enhances overall conversion efficiency and hydrogen recovery. Such coupling also facilitates the utilization of complex waste streams (Srivastava et al. 2024).

Advances in metabolic engineering, microbial consortia design, and process intensification are expanding the scope of waste valorization for biohydrogen production. Pretreatment strategies tailored for specific waste types, coupled with valorization of co-products, play a critical role in improving process economics. Furthermore, integration of biohydrogen routes into biorefineries provides a sustainable platform for waste management, bio-hydrogen production, and resource recovery.

The wet organic wastes can be processed using biological methods such as dark fermentation, where anaerobic bacteria break down complex organic compounds, producing carbon dioxide, hydrogen, and volatile fatty acids (Ghimire et al. 2015a). The process is straightforward, economical, and compatible. This also provides decentralized waste treatment facilities. The photo-fermentation process employs photosynthetic bacteria to further process the by-products of dark fermentation under light (Gupta et al. 2024). Although these methods are still in their early stages on a commercial level, they provide energy-efficient substitutes that are especially well-suited for wet organic streams such as sewage sludge and food processing waste. The waste-to-hydrogen systems offer substantial advantages from an environmental perspective. This technology can achieve negative carbon emissions by combining with CCS technologies (Shu et al. 2023). However, to determine the true environmental and economic performance of these technologies, LCA is necessary to evaluate feedstock logistics, emissions, and by-product management (Chen et al. 2025). The waste-to-hydrogen systems could be essential to the shift to a low-carbon, circular economy with continued technological advancements and benevolent regulatory frameworks. Table 1 depicts the literature on transforming biomass into biofuel.

## Smart feedstocks and microbial innovations

### Agricultural residues, food waste, and industrial biomass for bio-hydrogen production

Agricultural residues, food wastes, and industrial biomass serve as smart feedstocks for biohydrogen production due to their renewable nature, abundant availability, and high organic content. These materials, often considered as waste by-products from food and agricultural industries, offer a sustainable and cost-effective substrate for microbial conversion into hydrogen (Melikoglu and Tekin 2024; Yasin et al. 2013). By valorizing these agro-wastes, the approach addresses waste management challenges, reduces environmental pollution, and promotes a circular bioeconomy. Moreover, these feedstocks do not compete with food crops or require additional arable land, making them fitting for sustainable energy production. Their biochemical characteristics favour efficient microbial fermentation and photobiological hydrogen generation, thus constituting a “smart” choice for renewable bioenergy solutions (Ahmed et al. 2021; Hallenbeck and Ghosh 2012; Zhang et al. 2020).

### Microbial processes producing bio-hydrogen

**Fig. 2:**
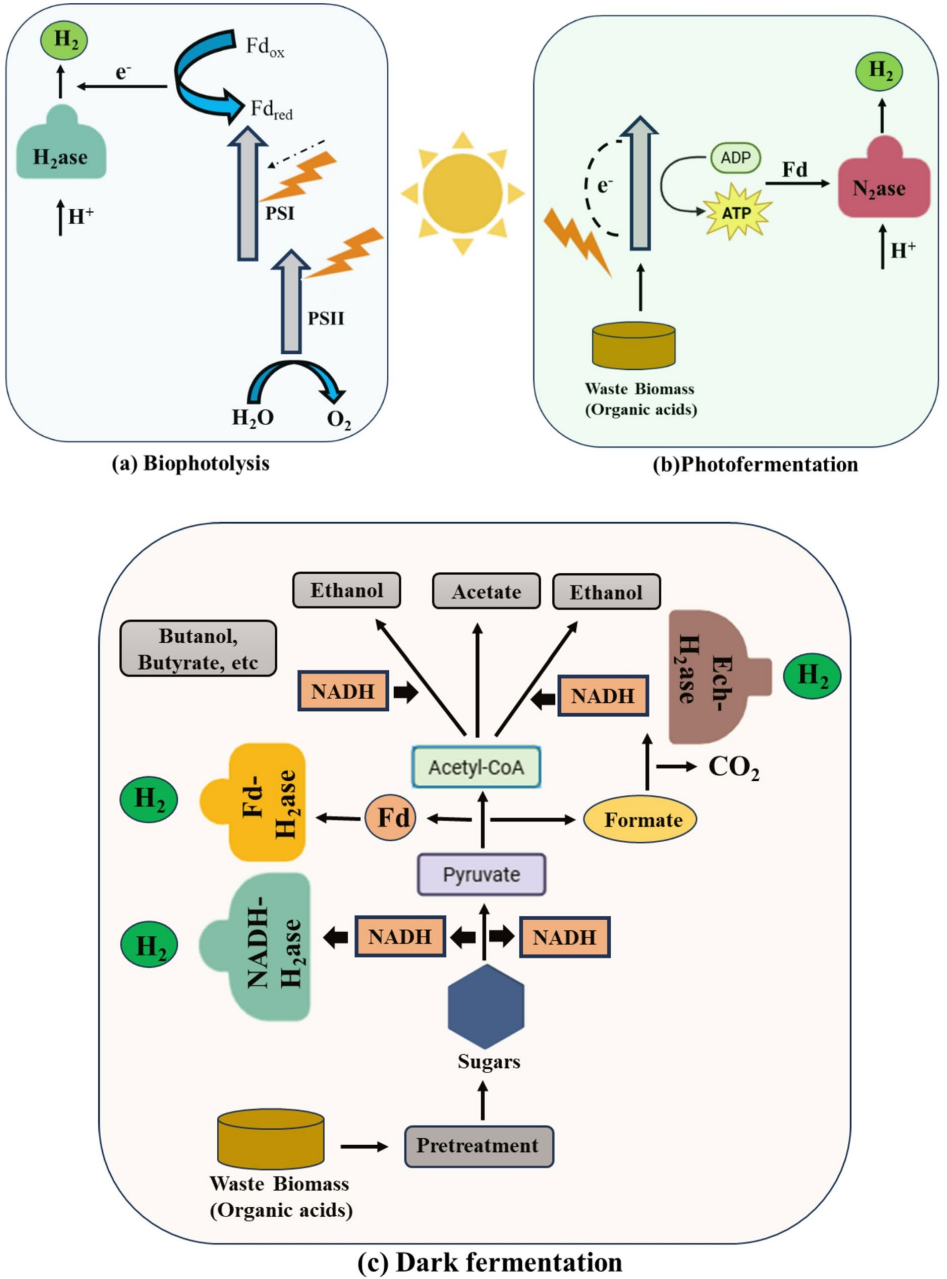
Schematic representation of **a** bio-photolysis, **b** photofermentation and **c** dark fermentation process for bio-hydrogen formation

Bio-hydrogen production refers to the metabolic activity of certain bacteria that degrade organic matter under anaerobic or facultative anaerobic conditions. Bio-hydrogen production refers to hydrogen generation by microbial metabolism via three major metabolic pathways, as illustrated in Fig. 2, namely (1) Bio-photolysis, (2) Fermentation, and (3) electrochemical processes (Ahmed et al. 2021).

#### Biophotolysis

Biophotolysis, both direct and indirect, biologically generates hydrogen fuel using sunlight and water via photosynthetic green microalgae and cyanobacteria. In direct biophotolysis, green microalgae such as *Chlamydomonas reinhardtii* use Photosystem II (PSII) to split water molecules, releasing electrons that travel through Photosystem I (PSI) and ferredoxin (Fd) to the oxygen-sensitive [FeFe]-hydrogenase enzymes. These enzymes catalyze the reduction of protons to molecular hydrogen (H_2_), dependent on efficient electron transfer and water splitting (Kong et al. 2010). Reactor configurations involve photobioreactors, often flat-panel or tubular, designed to optimize light capture, gas exchange, and maintain ambient temperatures near neutral pH to enhance enzyme activity while mitigating oxygen inhibition (Hallenbeck and Ghosh, 2012; Healey, 1970). *Chlamydomonas moewusii* uses a similar pathway but routes electrons from oxidative carbon metabolism directly through PSI, bypassing PSII, thereby reducing oxygen toxicity. It also produces hydrogen under dark conditions by metabolizing intracellular carbon reserves via fermentation metabolism combined with residual hydrogenase activity.

Indirect biophotolysis, exhibited by cyanobacteria like *Anabaena variabilis*, separates oxygen evolution and hydrogen production both temporally and spatially. In this two-step mechanism, photosynthesis via PSII and PSI fixes CO_2_ and generates organic substrates such as carbohydrates and oxygen. Subsequently, under anaerobic or microoxic conditions, stored substrates undergo fermentative metabolism, producing reducing equivalents that activate ferredoxin-dependent nitrogenase enzymes. Nitrogenase catalyzes proton reduction to hydrogen gas using ATP and reducing power. Mutant strains with inhibited hydrogen uptake and elevated nitrogenase expression under nitrogen deprivation further increase hydrogen evolution (El-Dalatony et al. 2020a; Sveshnikov et al. 2006). Reactors for indirect biophotolysis are designed to enforce microoxic or anaerobic environments, often using staged light and dark cycles or spatial separation of reaction chambers, optimizing nitrogenase performance and mitigating oxygen effects.

Green algae like *Chlamydomonas reinhardtii* produce hydrogen through water splitting but generate oxygen, which inactivates oxygen-sensitive hydrogenase enzymes and poses safety risks (Kong et al. 2010; Oh et al. 2024). Cyanobacteria such as *Anabaena variabilis* generate hydrogen via nitrogenase during nitrogen fixation, a metabolically costly process that limits yield (El-Dalatony et al. 2020a; Sveshnikov et al. 2006). The low solar-to-hydrogen conversion efficiency and extensive photobioreactor area required challenge economic viability (Gupta et al. 2024; Show et al. 2012). Catalyst strategies for these processes include genetic engineering approaches targeting [FeFe]-hydrogenase and nitrogenase genes to enhance enzyme activity and oxygen tolerance. Immobilisation techniques and co-culturing with complementary fermentative bacteria further improve volumetric productivity and substrate utilisation. Advances in photobioreactor design addressing light penetration, scaling issues, and gas–liquid mass transfer are critical to boosting solar-to-hydrogen conversion efficiencies and realizing commercial feasibility.

#### Fermentation

In photofermentation, photosynthetic non-sulphur (PNS) bacteria such as *Rhodobacter sphaeroides* utilize photons from solar light in photobioreactors designed to optimize light penetration, mixing, and gas exchange, typically using flat-panel or tubular configurations (Ahmed et al. 2021; Hallenbeck and Ghosh, 2012). These bacteria employ the tricarboxylic acid (TCA) cycle to metabolize organic substrates, often volatile fatty acids from dark fermentation, producing CO_2_, protons, and electrons. Light energy drives nitrogenase, the primary enzyme catalyzing hydrogen gas production by reducing protons to H_2_, simultaneously fixing nitrogen during ammonia synthesis (El-Dalatony et al. 2020a; Omer et al. 2025). Optimal conditions for photofermentation include 25–35 °C temperature, near-neutral pH (6.8–7.5), and light intensities of 2000–3000 lx (Hallenbeck et al. 2009).

Although photofermentation boasts high substrate conversion efficiency and hydrogen content typically reaching 58%, challenges such as slow production rates, sensitivity to light intensity, and costly reactor requirements limit scalability. Strategies enhancing yield and productivity include immobilization of bacteria, genetic engineering for nitrogenase overexpression, and co-culture techniques (Azwar et al. 2014; El-Dalatony et al. 2020a). Meanwhile, dark fermentation is a light-independent process carried out by anaerobic bacteria like *Clostridium butyricum* and *Enterobacter aerogenes* in batch or continuous stirred tank reactors (CSTR) (Mona et al. 2020; Rajesh Banu et al. 2020). This process metabolizes substrates such as agricultural, food, or industrial waste under acidic pH (5.5–6.5) and mesophilic temperatures (30–37 °C), with rigorous headspace hydrogen removal to alleviate product inhibition. Biohydrogen is produced via two main pathways: catabolic breakdown of formic acid through formate hydrogen lyase and re-oxidation of NADH via [NiFe]-hydrogenase, while pyruvate oxidation by pyruvate ferredoxin oxidoreductase (PFR) mediates central metabolism (El-Dalatony et al. 2020a).

Dark fermentation typically produces hydrogen at rates between 100–220 mL H_2_/L/h, with yields up to 4 mol H_2_/mol glucose (Table 2); however, hydrogen purity remains moderate (40–60%), often contaminated by CO_2_ and other gases (Mona et al. 2020). Process enhancements include metabolic pathway engineering via CRISPR-Cas9 for hydrogenase overexpression, immobilized biocatalysts to increase cell density, and optimized pH and substrate feed strategies (Azwar et al. 2014; Husaini et al. 2023).

**Table 2:** Roles of some major microorganisms contributing to bio-hydrogen production

Process	Microorganism	Role and highlights	Reactor type and conditions	Typical H_2_ yield/productivity details	References
Direct biophotolysis	*Chlamydomonas reinhardtii*	Photosynthetic water splitting in PSII, PSI, and hydrogenase enzyme	Photobioreactor (flat-panel); 25–30 °C; pH 7.0; light intensity ~ 1500 lx	1–2 mol H_2_/mol substrate	(Kong et al. 2010; Oh et al. 2024)
	*Chlamydomonas moewusii*	Dark hydrogen production via fermentation pathways and residual hydrogenase activity	Dark anaerobic bioreactor; 25–30 °C; pH 6.5–7.0	≤ 1 mol H_2_/mol substrate	(Healey, 1970)
Indirect biophotolysis	*Anabaena variabilis*	Nitrogen-fixing cyanobacteria; oxygen protection through temporal separation	Photobioreactor; 28–32 °C; pH 7.0; light intensity 2000 lx	2–4 mol H_2_/mol substrate	(Sveshnikov et al. 2006)
	*Synechocystis sp.*	ATP-driven hydrogenase activity	Photobioreactor; 25–30 °C; pH 7.0	0.02 mol H_2_/mol substrate	(El-Dalatony et al. 2020b)
	*Rhodobacter sphaeroides*	Photoheterotrophic bacteria metabolizing VFAs	Photobioreactor; 28–32 °C; pH 6.5–7.5; light intensity 2000 lx	~ 4–6 mol H2/mol acetate; 58% H_2_ content	(Gupta et al. 2024)
	*R. sulfidophilus*	Acid-tolerant, high-temperature; H2 production from acetate	Photobioreactor; acidic pH 5.5–6.5; 30–35 °C	3.59 ± 0.11 mol H_2_/mol acetate	(Cai and Wang, 2013; Gupta et al. 2024)
	*R. capsulatus*	Metabolic flexibility for variable substrates	Photobioreactor; 28–32 °C; pH 6.5–7.5; optimized conditions	5.5 ± 0.15 mol H_2_/mol glucose	(Ghosh et al. 2012; He et al. 2005)
Photo- fermentation	*Enterobacter aerogenes*	Facultative anaerobic fermenter	Anaerobic batch or CSTR; 30–37 °C; pH 5.5–6.5	2 mol H_2_/mol hexose	(Batista et al. 2018)
	*Escherichia coli*	Formate hydrogen lyase pathway	Anaerobic batch; 37 °C; pH 6.0	~ 0.96 mol H_2_/mol glucose	(Fan et al. 2009; Reith et al. 2003)
	*Clostridium beijerinckii*	Acetate-dominant fermentation	Anaerobic batch; 37 °C; pH 5.5–6.0	2.54 mol H_2_/mol glucose	(Hu et al. 2013; Humphreys et al. 2023)
	*Clostridium butyricum*	Efficient hydrogen producer near theoretical max	Anaerobic batch; 30–37 °C; pH 5.5–6.5	Up to ~ 4 mol H_2_/mol glucose	(Ntaikou et al. 2010)
Dark-fermentation	*Thermotoga maritima*	Hyperthermophilic fermentation	Anaerobic batch; 80–85 °C; neutral pH	4 mol H2/mol glucose	(Omer et al. 2025)
	*Thermoanaerobacterium thermosaccharolyticum*	Thermophilic carbohydrate fermenter	Anaerobic batch; 60–65 °C; pH 6.0	2.4 mol H_2_/mol glucose	(Omer et al. 2025; Ueno,’ et al. 2001)
	*Pyrococcus furiosus*	Extremophile hydrogen producer	Anaerobic batch; 95–100 °C; acidic to neutral pH	~ 3–4 mol H_2_/mol substrate	(Reith et al. 2003; Schut et al. 2012)
Microbial Electrohydrogenesis Cells (MECs)	*Shewanella oneidensis*	Electrogenic lactate oxidizer; cytochrome-mediated electron transfer	Dual-chamber MEC; 25–35 °C; pH 6.5–7.0; 0.6–1.0 V applied voltage	1.5–2.0 m^3^ H_2_/m^3^ reactor/day	(Rosenbaum et al. 2010)
	*Geobacter sulfurreducens*	Direct electron transfer via conductive pili and cytochromes	Dual-chamber MEC; 30–35 °C; pH 6.5–7.0; 0.6–1.0 V applied voltage	Up to 3.4 m^3^ H_2_/m^3^ reactor/day	(Fessler et al. 2023; Geelhoed and Stams, 2011)
Microbial fuel cells (MFCs)	*Thiobacillus ferrooxidans*	Iron-oxidising biofilm former; enhances system stability	MFC; 25–30 °C; pH 6.5–7.5	Power density up to 500 mW/m^2^	(Ulusoy and Dimoglo, 2018; van Alin et al. 2023)
	*Shewanella putrefaciens*	Riboflavin-mediated electron transfer	MFC; 25–30 °C; pH 6.5–7.5	Power density up to 700 mW/m^2^	(Pandit et al. 2014)
	*Aeromonas hydrophila*	Pili-based electron transfer, regulated by quorum sensing	MFC; 25–30 °C; pH 6.5–7.5	Power density ~ 250 mW/m^2^	(Castro et al. 2014)

#### Hybrid bio-hydrogen and synthetic biology for enhanced hydrogen yields

It is widely speculated that a two-step or hybrid biological hydrogen production method combining dark fermentation and photofermentation is essential to achieving sustainable and economically viable biohydrogen production (Trchounian et al. 2017). This hybrid technique leverages the advantages of each method while overcoming their individual limitations (Benemann, 1996). Photosynthetic non-sulfur (PNS) bacteria such as *Rhodobacter sphaeroides* metabolize volatile fatty acids (VFAs), the main soluble products generated during dark fermentation, breaking down energy barriers by utilizing sunlight to further convert these substrates into hydrogen (Fang et al. 2006; Kim et al. 2006).

**Fig. 3:**
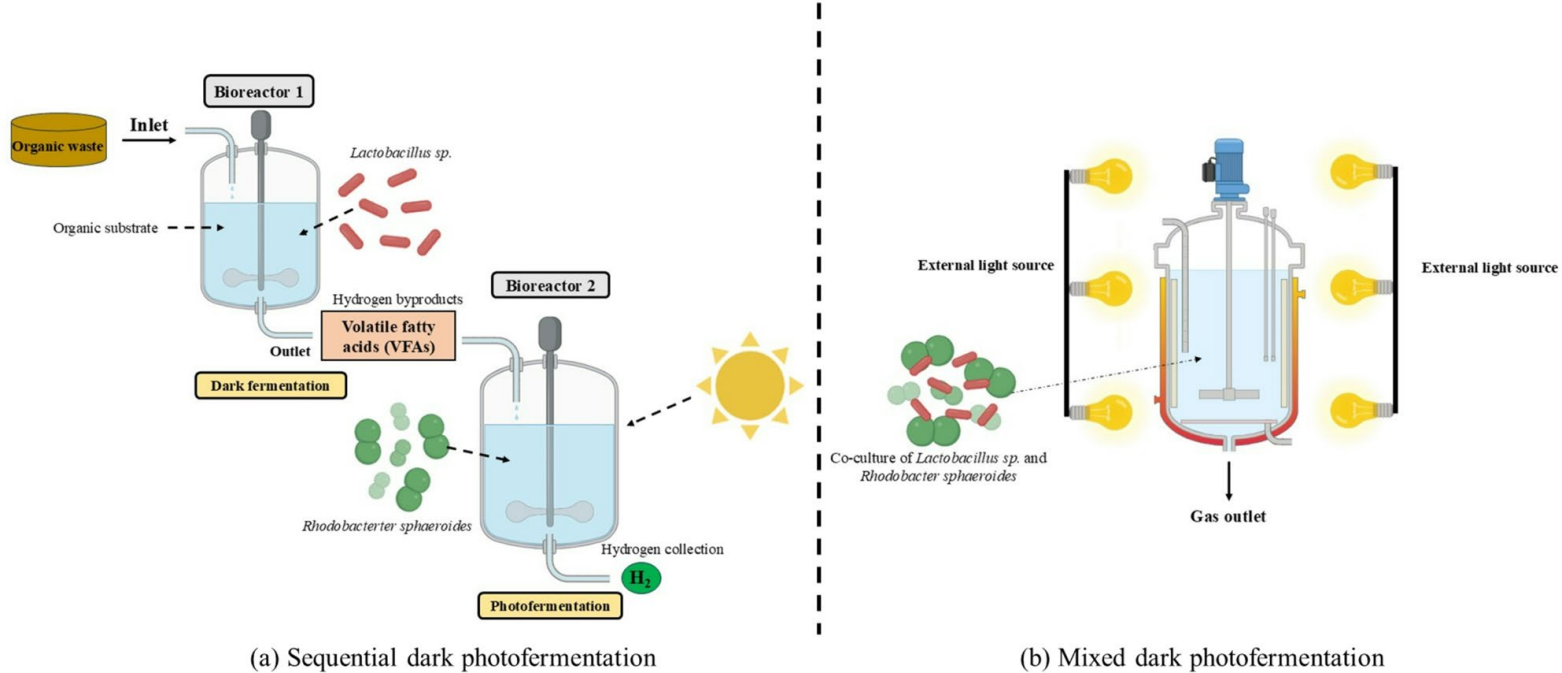
Schematic representation of the hybrid-mode of bio-hydrogen production, representing the **a** sequential dark photofermentation and **b** mixed dark photofermentation

In the typical two-step sequential dark-photo system (Fig. 3a), organic substrates are first decomposed in a dark fermentation bioreactor operated by bacteria such as *Clostridium butyricum* and *Lactobacillus sp.* at mesophilic temperatures (~ 35 °C) and acidic pH (5.5–6.5), producing hydrogen and VFAs. The VFAs are then subjected to photofermentation in a second, illuminated bioreactor operated by PNS bacteria like *Rhodobacter sphaeroides*, under near-neutral pH (6.8–7.5), temperature of 28–32 °C, and light intensities around 1500–3000 lx (Azwar et al. 2014; Rai and Singh, 2016). This arrangement allows continuous hydrogen generation by harnessing dark fermentation at night and photofermentation during the day, with hydraulic retention times typically from 12–24 h for both reactors. Although sequential systems incur higher capital costs due to two reactor units, they offer improved hydrogen yields. The mixed dark-photo system (Fig. 3b), in contrast, co-cultivates dark- and photo-fermentative bacteria like *Lactobacillus sp.* and *Rhodobacter sphaeroides* together in a single illuminated photobioreactor under continuous or semi-continuous operation. While this reduces hardware costs and simplifies operation, microbial competition and accumulation of unutilized VFAs often limit hydrogen yields (Asada et al. 2006; Fang et al. 2006). Metabolic and genetic engineering strategies are increasingly applied to enhance biohydrogen production efficiency. CRISPR-Cas9 gene editing, for example, is used to overexpress [FeFe]-hydrogenase genes while repressing competing pathways through targeted interference of [NiFe]-hydrogenase and fermentation genes such as ldh, adh, and spo0A. These molecular interventions also aim to improve microbial tolerance to inhibitory fermentation byproducts and facilitate lignocellulosic biomass degradation by integration of related enzymatic genes (El-Dalatony et al. 2020a; Husaini et al. 2023).

#### Electro-microbiological systems: microbial fuel cells and microbial electrolysis cells for electricity generation

**Fig. 4:**
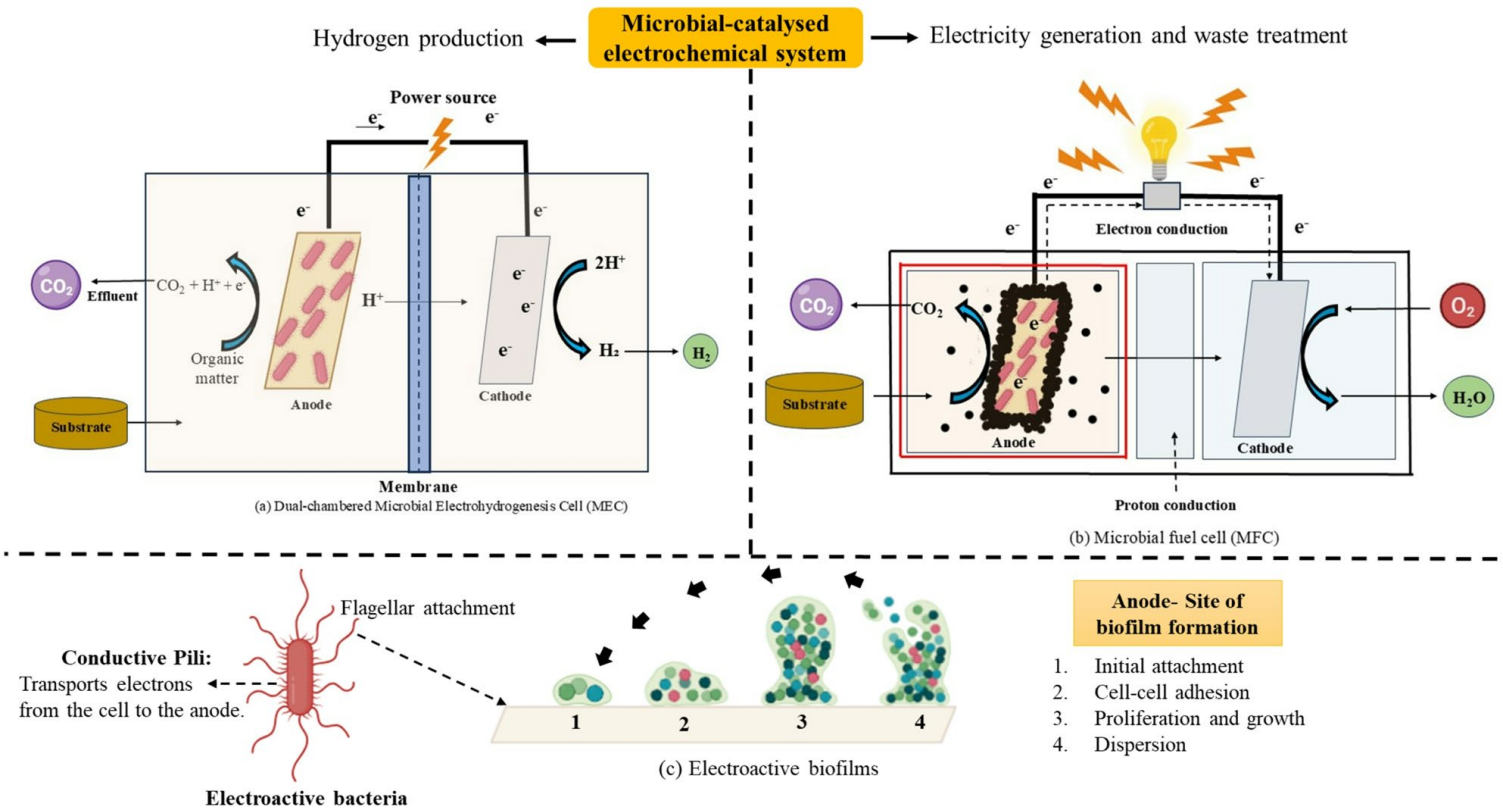
Schematic representation of microbial electrochemical system depicting the **a** MEC, **b** MFC and the **c** Electroactive biofilm develops at the anode, where bacteria respire electrochemically and facilitate their attachment to the anode surface

Microbial Electrohydrogenesis Cells (MECs) and Microbial Fuel Cells (MFCs) utilize specific electroactive bacteria capable of transferring electrons to solid electrodes (as shown in Fig. 4), enabling biohydrogen production and electricity generation. These systems oxidize organic substrates or CO_2_, producing protons and electrons using electroactive microorganisms like microalgae, *Geobacteraceae* (Jayabalan et al. 2020), and other bacteria listed in the Table 3 in context to MECs. To create molecular hydrogen (H^+^), electrons are moved to the anode, travel through an external circuit, and reduce protons at the cathode (Gautam et al. 2023). The mechanism is illustrated in Fig. 4. In MECs, *Shewanella oneidensis* harnesses L-lactate as an electron donor and transfers electrons to electrodes and metal oxides via outer membrane cytochrome c proteins, facilitating extracellular electron transfer (EET). This is augmented by the release of (ribo)flavins, which act as electron shuttles to external acceptors (Rosenbaum et al. 2010).

**Table 3:** Type of nanomaterials used in bio-hydrogen production and their energy efficiency

Type of nanomaterial	Source of nanomaterial	Energy efficiency rate	Scale-up application	Route	H_2_ yield	Reactor type	Key advantages	Technical challenges	References
Platinum catalysts and carbon nanotubes	Chemical precipitation	~ 3.5% under UV, increases with metal doping	Moderate scalability; cost-effective but photocorrosion prone	Photo-fermentation	2.1–2.5 mol H_2_/mol substrate	Quartz Tubular Reactor	Cheap, enhances electron mobility, boosts light absorption	Photocorrosion; reduced stability under long illumination	(Wang et al. 2012)
Gold (Au) Nanoparticles (Plasmonic)	Colloidal/green synthesis	Enhances photoactivity by ~ 20–30%	High cost limits scale-up, suitable for hybrid catalytic systems	Hybrid photocatalysis + photo-fermentation	20–30% increase over control (typically 2.8–3.2 mol H_2_/mol substrate)	Plasmon-enhanced photobioreactor	Strong plasmonic enhancement, improves visible-light harvesting	High cost, particle aggregation, requires stabilizers	(Hou and Cronin 2013)
Magnetic Iron Oxide (Fe₃O₄) Nanoparticles	Co-precipitation from iron salts	~ 12% when used as electron relay	Easily recoverable via magnet; scalable with fermentation	Dark fermentation	1.8–2.3 mol H_2_/mol glucose equivalent	Anaerobic stirred tank reactor	Enhances electron transfer; reduces fermentation lag phase; reusable	Possible inhibition at high doses; impacts microbial community	(Fernandes et al. 2015)
CdS Quantum Dots	Chemical synthesis/biotemplating	Up to 25% quantum efficiency (visible light)	High photocatalytic efficiency but toxicity restricts environmental scale	Photo-driven microbial–nanoparticle hybrid	3.0–3.6 mol H_2_/mol substrate (hybrid)	Hybrid photocatalytic–biological system	Excellent light absorption; efficient charge separation; boosts photobiological activity	Cadmium toxicity; disposal concerns; needs encapsulation	(Rautengarten et al. 2016)

Likewise, *Geobacter sulfurreducens* employs conductive pili (often termed microbial nanowires) and outer membrane c-type cytochromes (such as the Pcc pathway) to transfer electrons directly to anodes with high efficiency (Geelhoed and Stams, 2011). In MFCs, *Thiobacillus ferrooxidans* oxidizes Fe^2+^ via outer membrane enzymes, facilitating electron transport that enhances biofilm formation, system stability, and power generation, achieving power densities up to 500 mW/m^2^ (Ulusoy and Dimoglo, 2018). *Shewanella putrefaciens* delivers electrons to the anode through both direct contact and riboflavin-mediated pathways, essential for power generation improvements, reaching power densities up to 700 mW/m^2^ under optimized conditions (Pandit et al. 2014). *Aeromonas hydrophila* mediates electron transfer during anaerobic respiration through conductive pili nanowires regulated by quorum sensing, contributing to comprehensive microbial energy transmission and community stability, with power densities around 250 mW/m^2^ (Castro et al. 2014).

Typical operational parameters for these bioelectrochemical reactors include mesophilic temperatures (25–37 °C), neutral to slightly acidic pH (6.5–7.0), and voltage application for MECs around 0.6–1.0 V to facilitate proton reduction at cathodes. Reactor designs prioritise maximising biofilm formation on carbon-based electrodes, optimizing electron transfer pathways via biofilm engineering, and employing conductive materials to enhance current density and operational stability (Geelhoed and Stams, 2011; Rosenbaum et al. 2010). Additionally, increasing power output and efficiency remain essential for practical applications. Furthermore, comprehensive characterisation of microbial consortia, their syntrophic interactions, and adaptive responses to environmental fluctuations is necessary to optimise system stability. Advancements in real-time biosensing, process control, and bioinformatics-driven modelling could improve system robustness and operational efficiency (Oh et al. 2010).

## Role of nanotechnology and catalysts in enhancing hydrogen yield

### Nano-metal catalysts for microbial hydrogen production

Recent advances in bio-hydrogen production have been driven by the integration of nanoscale catalysts that overcome the intrinsic limitations of microbial electron transfer (Parsa et al. 2025). In particular, Titanium dioxide (TiO_2_), Magnetite (Fe₃O₄), and graphene-based nanomaterials have each demonstrated unique advantages in stimulating microbial consortia to evolve hydrogen more efficiently, while also offering routes to catalyst recovery and system stability (Mohd Jamaludin et al. 2023).

TiO_2_ nanoparticles leverage their well-known photocatalytic properties to enhance light-driven hydrogen evolution in hybrid bio-photoreactors. Mesoporous TiO_2_ nanofibers with oriented anatase nanocrystals, prepared via vapothermal transformation of titanate precursors at 150 °C, exhibit twice the hydrogen generation rate of benchmark P25 (photocatalyst) under identical irradiation and pollutant-degradation conditions (Hou et al. 2015). The aligned crystal domains facilitate rapid charge transport to microbial cells, and the mesoporosity enhances substrate diffusion, enabling simultaneous water reduction and contaminant oxidation in cascading oxic-anoxic processes (Cheng et al. 2012). When combined with gold nanoparticles and carbon nanotubes in a continuous‐flow microfluidic reactor, TiO_2_ hybrids further boost hydrogen output by up to 20-fold relative to pure TiO_2_, as synergistic charge separation and light absorption improve electron delivery to microbial hydrogenases (Cheng et al. 2012). Moreover, TiO_2_/Escherichia coli bio-hybrid systems under visible light have achieved a twofold increase in hydrogen yield at 2000 W/m^2^, with stability over multiple light incubation cycles and effective use of natural sunlight, underscoring TiO_2_’s role as a versatile electron mediator (Hou et al. 2015; Zhang et al. 2018).

Fe₃O₄ nanoparticles serve a dual function in dark-fermentation bio-hydrogen systems by both facilitating redox reactions and enabling facile magnetic recovery. Green-synthesized Fe₃O₄ from water hyacinth extract (20 mg/L) increased hydrogen yields by 23.5%, reaching 83.2 mL H_2_/g substrate, through upregulation of hydrogenase and formate-hydrogen lyase genes in *Klebsiella sp*. and a 2.1-fold enhancement of enzyme activity (Zhang et al. 2021). In *Enterobacter cloacae* fermentations, *Syzygium cumini* derived iron nanoparticles (100 mg/L) doubled the molar yield of H_2_ per mole of glucose versus ferrous sulphite (FeSO₄), highlighting the importance of nanoparticle bioavailability and surface chemistry (Nath et al. 2015). In scaled‐up expanded granular sludge bed reactors, 40–60 nm Fe₃O₄ NPs at 50 mg/L shifted the community from butyrate-type to ethanol-type fermenters, yielding 4.95 L H_2_/day and establishing conductive chains that accelerate interspecies electron transfer (Zhang et al. 2020).

Graphene-based materials capitalize on exceptional conductivity and surface area to strengthen MECs and bioelectrochemical reactors. A graphene-modified biocathode achieved hydrogen rates of 2.49 m^3^ H_2_/m^3^ reactor day with an electron recovery efficiency of 89% at -1.1 V (vs. Ag/AgCl), outperforming unmodified cathodes by nearly threefold and matching platinum catalysts in current density (Cai et al. 2016). When engineered into Nickel Cobaltite (NiCo_2_O₄)-graphene nanocomposites on nickel foam treating sugar-industry wastewater, cathodic hydrogen recovery reached 27.9% and overall H_2_ generation rose to 0.14 L/day—3.2 times that of bare foam-while maintaining 58.1% COD removal (Jayabalan et al. 2020). Biosynthesized Pd–Ag/rGO hybrids also demonstrate how graphene supports highly dispersed metal nanoparticles for rapid catalytic turnover, suggesting translatability to microbial systems where robust electron transfer underpins sustained hydrogen evolution (Jayabalan et al. 2020).

Despite these successes, challenges remain in controlling nanoparticle aggregation, mitigating potential cytotoxicity at elevated dosages, and ensuring long‐term stability under continuous operation. Future research should prioritize the design of core shell structures or polymer coatings to stabilize particles, explore synergies across multiple nano-metal catalysts, and integrate plasmonic or enzyme-immobilized systems to further boost activity. By tailoring surface chemistry and reactor architectures, nano-metal catalysts will continue to play a critical role in scaling microbial bio-hydrogen processes toward commercial viability and carbon–neutral energy production.

The incorporation of nano particles as the catalysts in microbial hydrogen production involves multiple production pathways. In which, the acetate pathway**,** one mole of glucose reacts with water to yield two moles of acetic acid, two moles of carbon dioxide, and four moles of hydrogen (C_6_H₁_2_O_6_ + 2 H_2_O → 2 CH₃COOH + 2 CO_2_ + 4 H_2_). In the butyrate pathway, glucose is converted into butyric acid, carbon dioxide, and hydrogen with a lower yield of 2 mol H_2_/mol glucose (C_6_H_12_O_6_ → CH₃CH_2_CH_2_COOH + 2 CO_2_ + 2 H_2_). Hydrogen can also be generated through formate decomposition by formate hydrogen lyase (HCOOH → H_2_ + CO_2_) and by proton reduction via hydrogenase enzymes (2 H^+^ + 2 e^−^ → H_2_). The presence of nano‐metal catalysts enhances microbial electron transfer, thereby boosting hydrogen evolution. Photocatalytic reactions by TiO_2_ under UV/visible light start with photon absorption and charge separation (TiO_2_ + hν → TiO_2_ (e^−^ + h^+^)), followed by hole‐driven water oxidation (2 h^+^ + H_2_O →1/2 O_2_ + 2 H^+^) and electron‐mediated proton reduction (2 e^−^ + 2 H^+^ → H_2_).

Similarly, Fe₃O₄ nanoparticles participate in redox cycling, where Fe₃O₄ consumes electrons and protons to form Fe^2+^ and water (Fe₃O₄ + 2 e^−^ + 8 H^+^ → 3 Fe^2+^ + 4 H_2_O), and Fe^2+^ can recycle electrons back to microbes, regenerating Fe₃O₄. Finally, graphene scaffolds facilitate direct interspecies electron transfer (DIET), with graphene accepting electrons (G + 2 e^−^ → G(2^–^)) and then mediating proton reduction at the graphene–microbe interface to release hydrogen (G(2^–^) + 2 H^+^ → G + H_2_).

### Enzyme immobilization using nanomaterials for efficient bioconversion

To overcome the limitations of poor stability, short operational lifetime and difficulty of reuse, enzyme immobilization onto nanomaterials has emerged as a promising strategy. It also enhances the enzymatic routes for biohydrogen production. Recent studies show that nanoscale support, such as carbon-based nanomaterials, metal-oxide nanoparticles, magnetic nanoparticles, metal–organic frameworks (MOFs), and covalent organic frameworks (COFs), and hierarchical nanoflowers, provide very high surface area, tunable surface chemistry, and favorable microenvironments that improve enzyme loading, orientation, and catalytic persistence (Maity et al. 2023). Common immobilization methods used with nanomaterials include adsorption, entrapment or encapsulation, covalent attachment and cross-linked enzyme aggregates. When these techniques are combined with nanostructured carriers, they enhance thermal and pH stability, reduce proteolytic degradation and improve reusability (Tadesse and Liu 2025). This attribute is critical for continuous bio-hydrogen production. Magnetic nanoparticles enable facile recovery and reuse of immobilized enzymes through magnetic separation (Gama Cavalcante et al. 2024).

The bio-hydrogen production is supported by preservation and activation of hydrogen-producing enzymes and electron transfer between biological catalysts and electron donors or acceptors when conductive nanomaterials are used as support (Cui et al. 2022). In the case of practical integrations, most of the studies include immobilized enzyme cascades for rapid saccharification and subsequent enzymatic conversion to hydrogen precursors (Sheldon et al. 2021). Also, literature shows work on nanoparticle-stabilized pretreatment enzymes that increase fermentable sugars released from lignocellulosic biomass and immobilized hydrogenase on conductive nanostructures in bio-electrochemical systems that improve electron uptake and hydrogen production rates (Dutta et al. 2023). To improve enzyme targets, several experiments report multi-fold improvements in hydrogen production rates.

The major challenges are the cost of nanomaterials and immobilization, the toxicity of nanoparticles to microbial catalysts, the leaching of the enzyme over time, and mass transfer limitations in dense immobilized matrices. There is a gap in the literature on life cycle and techno-economic analyses and regulatory concerns over engineered nanomaterials in large bioprocesses. The standardization of the immobilized matrix is necessary to compare studies and guide industrial translation.

The application of nanomaterials in enzyme immobilization has emerged as a trending technique for enhancing bioconversion processes, particularly in the sustainable production of hydrogen. The enzymes are immobilized when they are attached to solid supports, which improves their stability, reusability, and catalytic efficiency (Maghraby et al. 2023). Nanomaterials, including carbon nanotubes, metal–organic frameworks (MOFs), mesoporous silica, graphene oxide, and magnetic nanoparticles, are ideal platforms for immobilizing hydrogenase and other redox-active enzymes involved in the production of bio-hydrogen (Zhao et al. 2025). These distinct physicochemical characteristics of the material include high surface area, tunable porosity, and exceptional biocompatibility (Mane et al. 2024). These nanostructures aid in preserving the structural integrity of the enzyme and facilitate efficient electron transport, frequently increasing resistance to heat and chemical denaturation (Prabhakar et al. 2024). For instance, immobilization of hydrogenase on carbon nanomaterials or gold nanoparticles has demonstrated increased catalytic activity and prolonged operational lifetime under anaerobic conditions (Prabhakar et al. 2024). Table 3 depicts the type of Nanomaterials Used in Bio-hydrogen Production and Their Energy Efficiency.

In summary, enzyme immobilization through nanomaterials is a high-potential route to enhance biohydrogen conversion. However, work should focus on low-cost, biodegradable supports, mitigation of nanoparticle toxicity, rigorous scale-up demonstration, and integrated process designs that combine pretreatment, immobilized biocatalysts, and electrochemical coupling, for commercialization. For sustainable biohydrogen production via enzyme-nanomaterial, connecting nanotechnology, enzymology, and process engineering is essential.

### Plasmonic and photocatalytic nanoparticles in photo-fermentation

The plasmonic and photocatalytic nanoparticles are essential for accelerating photo-fermentation processes by enhancing light absorption, charge separation, and general photocatalytic performance for hydrogen production (Abdelaal et al. 2024). The photosynthetic bacteria convert organic substrates into hydrogen when exposed to light (Afsar et al. 2011). However, photo-fermentation occasionally faces limitations because of its low quantum efficiency and poor light consumption. Plasmonic nanoparticles, such as gold (Au), silver (Ag), and copper (Cu), can significantly improve light harvesting by increasing the absorption range of the device and intensity of light through localized surface plasmon resonance (LSPR) (Mcoyi et al. 2024). The nanoparticles intensify electromagnetic fields and increase the activation of photosynthetic processes as light antennas.

Photocatalytic nanoparticles such as graphitic titanium dioxide (TiO_2_), carbon nitride (g-C₃N₄), and zinc oxide (ZnO), produce reactive electron–hole pairs when exposed to light (Ashwini et al. 2025). These substances may transfer photogenerated electrons to the bacterial cells when paired with photo-fermentative systems, enhancing hydrogenase activity and metabolic activity. Moreover, plasmonic and photocatalytic components, when combined with nanocomposites, exhibit synergistic effects, increasing the photonic and catalytic efficiencies. The stability and reusability of these nanoparticles allow for scalability and longer operation (Ashwini et al. 2025). However, concerns like the toxicity, aggregation, and environmental effects of nanoparticles necessitate careful consideration.

Studies have been reported on the implementation of gold–silica core–shell nanoparticles with *Rhodopseudomonas palustris* in near infrared illumination to achieve higher yield of hydrogen of 2.5-fold (Ji et al. 2021). The hydrogen production was improved and stabilized through hybrid dark or photo-fermentation reactors incorporating Fe₃O₄–TiO_2_ nanoparticles. Studies revealed that Photoreforming using Pt/TiO_2_ has been applied to fermentation effluents rich in volatile fatty acids, directly generating hydrogen from waste organics under illumination (Atilano-Camino et al. 2024). Collectively, these findings illustrate the potential of plasmonic and photocatalytic nanoparticles to augment microbial processes and improve solar-to-hydrogen efficiency. However, there are some challenges that need to be tackled in these techniques of biohydrogen production. Firstly, the interaction of nanoparticles and microbes should be carefully managed as it exhibits antimicrobial effects. Secondly, the maintenance of the stability and dispersion of nanoparticles within biological conditions is challenging. The third challenge is a varying integration strategy, as nanoparticles may disperse directly in cultures, immobilized on supports, or used in separate photocatalytic compartments. The immobilization strategies that preserve optical effects while allowing catalyst reuse will aid scalability. To increase overall hydrogen yields, hybrid systems coupling photo-fermentation with downstream photocatalytic photo reforming of effluents represent a pragmatic near-term approach. Thus, a promising new avenue for the production of sustainable bio-hydrogen is offered by the combination of plasmonic and photocatalytic nanomaterials in photo-fermentation, which offers increased efficiency and potential for commercial application. Table 4 depicts the integration of nanomaterials concept with microbial systems.

**Table 4:** Integrating nano materials with microbial activity for bio-hydrogen production

Nano material type	Type of microbial activity	Microbial kinetic rate	Hydrogen production efficiency (%)	Overpotential (mV)	Current density	Coulombic efficiency (%)	Operating conditions	References
Ni mesh cathode	MEC (mixed culture)	4.18 m^3^ H_2_/m^3^·d	89%	350–420 mV	6.8–7.5 A/m^2^	82–90%	pH 7.0; 30 °C; acetate feed	(Kadier et al. 2015)
MoSe_2_/3d-metal oxide hybrids	HER (abiotic–bio hybrid)	Not reported	0% (bio-H_2_ not quantified)	180–220 mV	10–15 mA/cm^2^	Not applicable	pH 1–2 (acidic HER system)	(Najafi et al. 2018)
Ni_2_P nanoparticles on carbon	MEC	0.29 L H_2_/L·d	0 (bio-only not specified)	250 to −00 mV	2.5–3.2 A/m^2^	65–72%	pH 7.2; room temperature	(Kim et al. 2019)
NiMoO₄ on Ni foam	MEC	0.12 L H_2_/L·d	12%	280–340 mV	1.8–2.1 A/m^2^	54–60%	pH 7; 30 °C	(Kim et al. 2019)
Cu_2_O and MoS_2_ nanoparticles	MEC (Makgeolli wastewater)	0.95–1.55 m^3^ H_2_/m^3^·d	0% (bio fraction not separately given)	260–330 mV	4.5–6.0 A/m^2^	70–78%	pH 6.8; 25–30 °C; brewery wastewater	(Wang et al. 2019)
NiO/Co₃O₄ on Ni foam	MEC	Not reported	0%	~ 300–360 mV	3.5–4.2 A/m^2^	60–68%	pH 7; acetate substrate	(Rani et al. 2021)
Ni–Co–P nanocomposite	MEC	Not reported	0%	~ 200–280 mV	7–10 A/m^2^	70–85%	pH 7; mesophilic	(Chaurasia et al. 2021)
Cu/NiMo composite	MES (*Cupriavidus necator* H16)	Not reported	~ 50% increase vs. control	~ 400–480 mV	5–8 mA/cm^2^	30–45%	CO_2_-fed MES system; pH 6.8	(Moon et al. 2024)

## Bio-hydrogen purification, storage, and utilization

### Advanced membrane technologies for bio-hydrogen purification

Bio-hydrogen produced via dark fermentation (DF), photofermentation, and related bioprocesses typically emerges as a low-pressure gas mixture containing 20–60 vol % H_2_, along with CO_2_, trace contaminants, and moisture. To meet fuel cell-grade specifications (> 99.99% H_2_) and for most industrial uses, purification is essential. Traditional techniques such as cryogenic distillation and pressure swing adsorption (PSA) are energy-intensive and often uneconomical at small–medium scales (Abdul Muin et al. 2021).

By contrast**,** membrane-based separation has emerged as an energy-efficient, scalable method. Polymeric membranes (e.g., polyimide, polysulfone) are mature and low-cost but suffer from limited selectivity (H_2_/CO_2_ separation factor ~ 3–5) and low thermal stability. To address these gaps, hybrid systems combining polymers with inorganic fillers—known as mixed matrix membranes (MMMs) show enhanced permeance and selectivity, hitting separation factors above 10 while retaining decent throughput.

Simultaneously**,** inorganic dense metal membranes**,** particularly palladium-based alloys, enable near-complete extraction of H_2_ due to Pd’s unique permeability for atomic hydrogen. These membranes achieve purities of 99.99% H_2_ but require high temperatures (> 300 °C) and pose cost and embrittlement issues (Lu et al. 2021). Low-cost ceramic and zeolite membranes (e.g., silica, alumina–zeolite composites) are being engineered as lower-temperature alternatives; these utilize molecular sieving to enhance separation efficiency through sub-nm pores, thermal resilience, and chemical robustness. A novel pathway is hollow-fiber membrane reactors that integrate gas-permeable membranes directly into fermentation vessels either submerged or side-stream enabling simultaneous hydrogen production and purification. This synergy shifts the fermentation equilibrium toward H_2_ via on-line removal, suppresses inhibition by microbial by-products, and yields higher H_2_ concentrations (up to 80 vol %) in permeate while increasing overall bio-hydrogen productivity (García-Depraect et al. 2025).

In recent years, the advent of 2D nanomaterial-enhanced membranes has opened new frontiers. Graphene oxide and carbon nanotubes, when embedded in polymer matrices or layered between ceramic supports, provide ultra-thin selective layers (~ tens of nm) with molecular sieving enhanced by surface functional groups and controlled interlayer spacing. These nanocomposite membranes combine high permeance rates (> 2000 GPU) with strong selectivity (H_2_/N_2_ > 100), showing promise for low-cost, high-throughput, small-footprint systems.

Nevertheless, practical implementation faces several challenges:

- **Fouling and stability**: Biomass particulates and extracellular polymeric substances can clog pores and reduce flux. Advanced pre-filters and hydrophilic coatings are being developed to mitigate this.
- **Permeability-selectivity Trade-off**: Higher throughput often leads to poorer separation, requiring careful optimization of material design via MMMs and hierarchical structures.
- **Scalability and manufacturing costs**: High-performance membranes using Pd or graphene are still expensive, though miniaturization and roll-to-roll deposition techniques are reducing production costs.
- **Durability under fermentation flux**: Chemical resistance to acidic/basic conditions, moisture cycles, and microbial interactions is essential for long-term operation.

### Metal hydride and graphene-based hydrogen storage systems

Efficient hydrogen storage remains a pivotal bottleneck in realizing a bio-hydrogen-based energy economy. Compressed gas and cryogenic methods are resource-intensive and suffer from safety limitations. Solid-state storage using metal hydrides offers a compelling alternative due to high volumetric energies, safety, and stable kinetics. Traditional hydrides (e.g., MgH_2_, LaNi_5_H_6_, NaAlH₄) can store up to 7–10 wt % hydrogen but face high desorption temperatures (> 300 °C), slow kinetics, and cyclic degradation (Mohammadi et al. 2022). Advances in nano-structuring have begun to lower these thermal and kinetic barriers. Reducing particle size to the nanoscale significantly enhances surface reactive sites and shortens diffusion paths, enabling faster kinetics and lower desorption temperatures. Notably, MgH_2_ nanoparticles stabilized within graphene-derived frameworks or carbon scaffolds have become a focus:

- Heat transfer is improved due to graphene’s thermal conductivity.
- Aggregation is minimized by graphene support.
- These composites (e.g., MgH_2_ rGO) demonstrate onset desorption near 100 °C and maintain ~ 1.5–2 wt % reversible capacity across multiple cycles (Li et al. 2015)

Graphene itself can act as a hydrogen adsorbent. Though pristine graphene exhibits weak physisorption (~ 0.1 wt %), Al-doped graphene can boost hydrogen uptake to ~ 5 wt % at near ambient conditions, owing to metal–hydrogen orbital interactions as suggested by DFT studies. Also, applying compressive strain (~ 6%) to graphene significantly increases H_2_ adsorption energetics, achieving theoretical capacities up to 9 wt % (Li et al. 2015). These breakthroughs point to the potential of tunable graphene-based adsorbents. Beyond physisorption, graphene–hydride nanocomposites offer synergy: graphene enhances physical properties (conductivity, mechanical strength), while the alloy/hydride provides high chemical storage capacity. Examples include MgH_2_ confined in graphene-pillared organosilica supports, which show dehydrogenation starting at ~ 50 °C with stable 1.62 wt % reversibility over multiple cycles (Comanescu 2023).

Still, key challenges remain for real-world implementation:

- **Storage capacity**: Composite systems achieve only 1–2 wt %, below DOE targets (~ 6 wt % for vehicular use). New nanostructured alloys (e.g., LiH, NaBH₄) are being evaluated.
- **Thermal management**: Hydrogen absorption is exothermic; release is endothermic. Effective thermal pathways via graphene scaffolds or heat-transfer materials are critical.
- **Scalability and cost**: High-quality graphene and nano-MH require controlled synthesis routes. Emerging production processes (chemical vapor deposition, waste-graphite upcycling) and AI-guided design are helping drive costs down.
- **Material durability**: Nanostructures can oxidize or sinter over cycles. Encapsulation within porous supports and protective coatings are current strategies.

Complementing nanocomposites, high entropy hydrides multi-element alloys with tailored lattice sites offer reversible hydrogen storage at near room temp with fast kinetics and low hysteresis, as demonstrated in Ti–Zr–Cr–Mn–Fe–Ni systems (Yartys et al. 2025). These materials show stable cycling (> 1000 cycles) and capacity comparable to LaNi_5_ with the added benefit of ambient operating conditions.

In summary, metal hydrides enhanced via nanotechnology and graphene scaffolding are revolutionizing hydrogen storage mode by attaining lower temperature operation, faster sorption rates, and safer storage forms. With ongoing innovation in nanocomposite engineering**,** material design using AI, and cost-effective synthesis, solid-state storage is closer to meeting DOE goals offering viable pathways for integrating storage into bio-hydrogen production sites and fuelling adoption in transportation, power backup, and grid stabilization roles.

### Direct bio-hydrogen utilization in fuel cells and industrial applications

Optimizing the sustainability of the bio-hydrogen value chain requires seamless integration of production, storage, and utilization. Direct use of purified bio-hydrogen in fuel cells and industrial settings enables distributed, low-carbon energy systems and aligns with circular economy objectives.

The sustainable utilization of bio-hydrogen represents a pivotal link in the hydrogen economy, where production, storage, and end-use must be efficiently aligned. Direct utilization of bio-hydrogen in fuel cells and industrial processes not only ensures a closed-loop, low-emission energy pathway but also amplifies the role of circular bio economy by valorising biological waste into a clean energy vector. As technological advancements enable higher purity bio-hydrogen through membrane purification and nanomaterial-enhanced processing, its deployment in power generation and manufacturing has become increasingly viable (Kayan et al. 2025).

Fuel cells represent a transformative technology for converting hydrogen into electricity with high efficiency and minimal emissions. Among the various fuel cell types, proton exchange membrane fuel cells (PEMFCs) are particularly well-suited for bio-hydrogen applications due to their compactness, low operating temperatures (~ 80 °C), and quick startup capabilities. However, PEMFCs require hydrogen of ultra-high purity (> 99.99%) to prevent catalyst poisoning, especially by CO and H_2_S (Sadeq et al. 2024). Recent advances in membrane-based purification, particularly using graphene-oxide and mixed-matrix membranes, have enabled bio-hydrogen derived from dark fermentation or photo fermentation to meet the purity thresholds required for PEMFC operation. Pilot demonstrations have successfully integrated membrane-purified bio-hydrogen with PEMFC stacks, achieving consistent power output ranging from 50 to 200 W with electrical efficiencies above 45% (Tellez-Cruz et al. 2021).

Solid oxide fuel cells (SOFCs) offer another avenue for bio-hydrogen utilization, especially where the hydrogen stream may contain impurities or be part of a synthetic gas mix. Operating at high temperatures (600–800 °C), SOFCs can tolerate certain contaminants and can directly utilize bio-hydrogen without extensive conditioning (Sharma et al. 2016). This makes them particularly attractive for applications where high-grade hydrogen purification may not be feasible or economical. SOFCs integrated with dark fermentation and downstream catalytic filters have shown electrical efficiencies exceeding 50%, especially when coupled with biogas streams containing H_2_ and CO_2_ (Saadabadi et al. 2019). The ability to co-generate heat further improves the system’s total energy efficiency, making it suitable for combined heat and power (CHP) applications in decentralized grids and agro-industrial setups (Sharma et al. 2016).

A particularly innovative approach lies in metal hydride fuel cells (MHFCs), which combine hydrogen storage and fuel cell functionality into a compact unit. In MHFCs, hydrogen is stored in a solid-state metal hydride matrix and is released through thermal activation to feed the fuel cell. These systems are inherently safe, operate at moderate temperatures (60–90 °C), and exhibit excellent cold-start characteristics (Gkanas et al. 2022). MHFCs have been tested in portable and backup power systems, delivering outputs of up to 1 kW with over 7,000 h of cumulative operational stability. When coupled with bio-hydrogen streams, especially from batch-fed fermenters, MHFCs provide a self-contained, emission-free energy solution that is particularly attractive for off-grid, military, or rural deployments (Gkanas et al. 2022).

Beyond power generation, bio-hydrogen holds significant promise in industrial applications. One of the primary industrial uses of hydrogen is in ammonia synthesis via the Haber–Bosch process. Traditionally, hydrogen used in this process is derived from natural gas through steam methane reforming, resulting in substantial CO_2_ emissions. Replacing fossil hydrogen with bio-hydrogen, especially when produced from agricultural residues or food waste, offers a low-carbon alternative for fertilizer production. Similarly, bio-hydrogen can serve as a clean reducing agent in hydrogenation reactions used in food processing, pharmaceuticals, and petrochemical refining. These industries demand high hydrogen purity and reliability, both of which are increasingly being met by advancements in membrane separation and nanomaterial filters (Sadeq et al. 2024).

In metallurgical applications, hydrogen is used as a reducing agent in iron and steel production. Direct reduction of iron ore using bio-hydrogen can significantly lower the carbon footprint of steel manufacturing. Decentralized mini-mills or small-scale foundries can especially benefit from local bio-hydrogen production units, enabling cleaner, more efficient processing without dependence on centralized hydrogen supply chains. Techno-economic studies have shown that integrating membrane-purified bio-hydrogen with modular hydrogenation units can reduce operational and capital expenditures by 15–30% compared to conventional supply models (Wang et al. 2021).

The integration of bio-hydrogen systems into the broader framework of circular economy offers additional value. For instance, co-located facilities using waste biomass for hydrogen production can create zero-waste ecosystems (Kayan et al. 2025). The by-product CO_2_ can be sequestered or utilized in algae cultivation, while waste heat from fuel cells can be used for process heating or feedstock drying. Metal hydride-based storage systems provide resilience and energy buffering, enabling continuous operation even during downtime in bio-hydrogen generation. These integrated systems are particularly well-suited for rural areas, agro-industrial clusters, and microgrid communities, where centralized energy infrastructure is limited (Li and Yao 2024). Despite these advantages, several challenges must be addressed to achieve widespread adoption. Ensuring hydrogen purity remains a key hurdle, particularly the removal of sulphur compounds and volatile organics that can poison fuel cell catalysts.

**Fig. 5:**
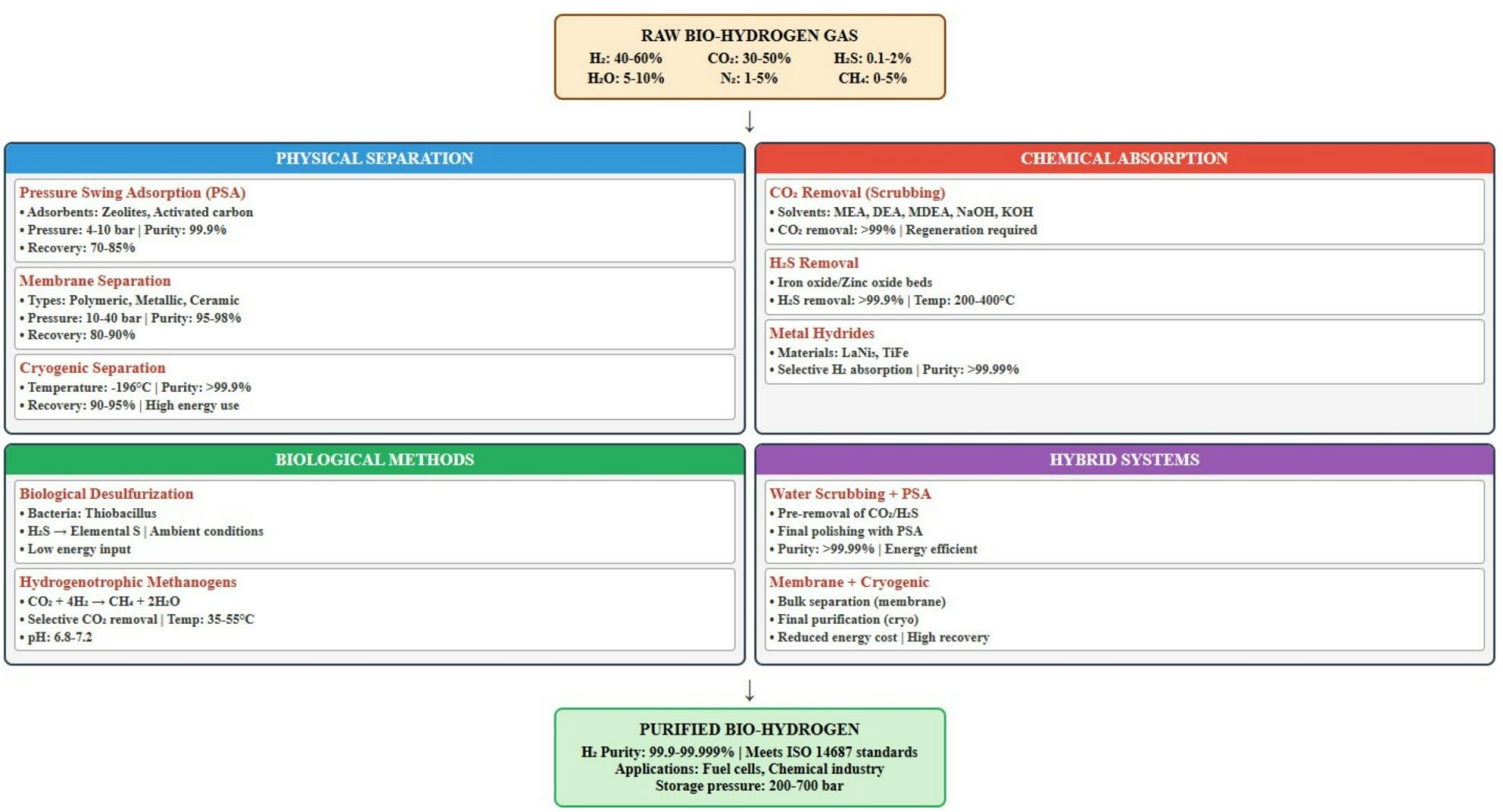
Schematic diagram of available purification systems for bio-hydrogen

Advanced nanostructured adsorbents and catalytic polishing units are being developed to provide in-line gas conditioning (Islam et al. 2024). Scalability and modularization are also crucial; as most current systems are lab- or pilot-scale. Developing standardized units that can deliver power from 100 W to 10 kW will be critical for broad deployment. Additionally, robust regulatory frameworks addressing storage, safety, and emissions will be essential to enable safe integration of hydrogen systems into industrial and community settings (Fathima et al. 2024). Cost competitiveness is another area of focus. While bio-hydrogen has the potential to become cost-effective especially when integrated into waste valorisation pathways reducing the costs of advanced materials such as graphene, hydride alloys, and nano-membranes remains essential. Leveraging economies of scale, local fabrication techniques, and AI-guided materials discovery may accelerate this cost reduction. Moreover, the potential for co-products such as organic acids, bioplastics, or fertilizers from the same fermentation streams enhances the economic viability of integrated bio-hydrogen systems (Islam et al. 2024). Figure 5 depicts the available purification systems for bio-hydrogen gas.

## Future outlook for research and development

### Summary of key findings

This comprehensive review has highlighted the pivotal role of bio-hydrogen in the global transition toward a sustainable and circular energy economy. A confluence of biological innovation, nanotechnology, and integrated waste-to-energy systems underscores the technological maturity and future promise of bio-hydrogen production. Renewable feedstocks such as agricultural residues, municipal solid waste, food processing by-products, and microalgae were found to be highly effective substrates, each offering distinct advantages in terms of availability, biodegradability, and conversion efficiency.

Innovations in microbial engineering including hybrid fermentation systems, extremophile consortia, and synthetic biology tools like CRISPR-Cas9 have significantly enhanced bio-hydrogen yield and stability. Furthermore, nanotechnology has emerged as a game-changer across all stages: from enhancing enzymatic and photobiocatalytic efficiency to improving hydrogen selectivity and sorption kinetics in advanced purification and storage systems. Mixed-matrix membranes, graphene-functionalized catalysts, and nanostructured metal hydrides have shown remarkable potential to overcome conventional bottlenecks such as low gas purity and high desorption temperatures.

The practical deployment of bio-hydrogen is currently limited by several technical, economic, and infrastructural challenges, including: (i) low hydrogen yields and production rates under real operational conditions, (ii) high cost and instability of biological systems, (iii) difficulties in large-scale storage, compression, and transportation due to hydrogen’s low energy density and diffusivity, (iv) limited compatibility of existing municipal and industrial infrastructures with hydrogen-based technologies, and (v) lack of clear regulatory frameworks and market incentives for bio-hydrogen integration. These barriers collectively hinder the transition from laboratory-scale research to full-scale applications in household, municipal, transport, and industrial sectors.

From a utilization perspective, the integration of bio-hydrogen into PEMFCs, SOFCs, and MHFCs has enabled clean, localized bio-hydrogen based power generation suitable for both stationary and mobile applications. Decentralized hydrogen units powered by waste feedstocks have proven particularly effective for rural electrification and microgrid deployments. In addition, the industrial relevance of bio-hydrogen in ammonia synthesis, green metallurgy, and chemical hydrogenation has expanded its appeal beyond the energy sector. Together, these findings affirm bio-hydrogen’s transformative potential across environmental, technological, and industrial domains.

### Future directions in sustainable bio-hydrogen research

To fully harness the promise of bio-hydrogen, interdisciplinary research and system-wide innovations must be strategically advanced. One critical direction involves the application of *omics* technologies genomics, transcriptomics, and metabolomics to unravel the metabolic fluxes of hydrogen-producing microbes. The data-driven optimization of metabolic pathways, in tandem with synthetic biology approaches, will facilitate the design of high-yield strains with resistance to substrate inhibitors and product toxicity.

Artificial intelligence and machine learning offer powerful tools for dynamic process control and predictive modeling in bio-hydrogen systems. AI can optimize fermentation parameters in real time, forecast microbial community shifts, and identify optimal nanomaterials or hybrid system designs based on feedstock variability and reactor conditions. Additionally, bioreactor innovation will be essential. Modular, multi-stage reactors equipped with embedded nanofiltration membranes and in-line monitoring can allow for continuous hydrogen recovery and real-time purification, thus improving efficiency and reducing downtime.

On the materials front, the rational design of multifunctional nanocomposites combining catalytic activity, thermal conductivity, and chemical durability is crucial. Bio-inspired nanomaterials and green synthesis methods should also be prioritized to align with sustainability principles. Furthermore, research should expand to the creation of integrated biorefineries where hydrogen production is co-located with carbon capture, nutrient recycling, and co-product recovery (e.g., volatile fatty acids, bioplastics).

Equally important are policy-aligned techno-socio-economic studies. These should address supply chain logistics, lifecycle emissions, hydrogen quality regulations, and waste feedstock availability. The development of modular systems for hydrogen production, storage, and utilization in community or industrial settings complemented by strong regulatory frameworks and public–private investment will determine the scalability and societal acceptance of bio-hydrogen technologies.

### Recommendations for industrial applications

To transition bio-hydrogen from laboratory feasibility to industrial scalability, specific strategies must be tailored to current infrastructure and sector-specific needs. Firstly, decentralized bio-hydrogen units utilizing locally available organic waste should be prioritized in agro-industrial clusters, food parks, and wastewater treatment facilities. These units can support on-site bio-hydrogen production, thus reducing dependence on fossil-fuel grids and offering an economically feasible alternative in regions with inadequate energy infrastructure.

Industries with high hydrogen demands such as chemical manufacturing, oil refining, and fertilizer production can retrofit existing hydrogen supply systems with purified bio-hydrogen sources. This may involve modifications in pressure systems, catalyst beds, or purification protocols, particularly when transitioning from steam methane reforming (SMR) to biological sources.

Fuel cell-based power generation offers another major application. PEMFCs powered by purified bio-hydrogen can serve as emission-free alternatives to diesel generators for backup power in hospitals, data centers, and industrial zones. Similarly, metal hydride-based fuel cells (MHFCs) provide a compact and reliable power source suitable for military, telecom, and off-grid locations. Coupling these systems with modular fermentation bioreactors can ensure uninterrupted hydrogen supply and safe storage.

In heavy industries such as steelmaking and cement production, bio-hydrogen can act as a low-carbon reductant. Pilot-scale demonstrations using hydrogen-based direct reduction of iron (DRI) have shown significant emissions savings. Integrating bio-hydrogen into these high-heat processes offers both environmental and economic advantages, particularly under carbon taxation regimes.

Additionally, industrial parks and SEZs can adopt circular economy models that integrate bio-hydrogen production with carbon utilization (e.g., algae cultivation), nutrient recovery, and bio-based co-product synthesis. Such integrated platforms will not only improve energy and material efficiency but also open up new revenue streams. Cost reduction remains essential for broader adoption. This can be achieved by leveraging economies of scale, adopting waste-derived feedstocks, and deploying locally sourced materials such as agricultural residues or algal biomass. Moreover, carbon credit mechanisms and green certifications should be tapped to enhance market competitiveness and policy compliance.

In summary, by aligning technical advancements with regulatory frameworks and economic incentives, bio-hydrogen can become a mainstream energy vector across sectors. Its integration into existing industrial operations, coupled with sustainable feedstock utilization and nanotechnology-enhanced processes, positions bio-hydrogen as a viable, circular, and climate-resilient alternative to fossil-based hydrogen systems.

## Conclusion

This comprehensive review demonstrates that bio-hydrogen production represents a paradigm shift toward sustainable energy systems, successfully integrating environmental remediation with clean bio-hydrogen production. The convergence of advanced microbial engineering, nanotechnology innovations, and circular economy principles has transformed bio-hydrogen from laboratory curiosity to commercially viable technology. Key findings reveal that renewable feedstocks, particularly agricultural residues and organic waste streams, offer abundant, cost-effective substrates that eliminate food-versus-fuel conflicts while enabling waste valorization. Nanotechnology integration has proven transformative, with nano-metal catalysts achieving up to 20-fold improvements in hydrogen production rates, while advanced membrane systems enable simultaneous production and purification with over 98% efficiency. Microbial innovations, including hybrid fermentation systems and genetic engineering approaches, have substantially enhanced yields and process stability.

The successful integration of bio-hydrogen into fuel cells and industrial applications validates its potential as a direct replacement for fossil-based hydrogen. However, challenges remain in scaling production, reducing costs, and developing robust regulatory frameworks. Future research priorities should focus on AI-driven process optimization, multifunctional nanocomposite development, and integrated bio-refinery concepts. Bio-hydrogen’s unique ability to bridge waste management and energy production positions it as a critical enabler of the circular economy, offering unprecedented opportunities for sustainable industrial transformation and climate change mitigation.

## Data Availability

All data, models or experimental frameworks used during the study are available from the corresponding author by request.
